# Pulsed Electric Field‐Assisted Recovery of Phenolic Compounds From Haskap (*Lonicera caerulea* L.) Pomace: Influence of Processing Parameters and Cell Disintegration

**DOI:** 10.1111/1750-3841.71292

**Published:** 2026-07-13

**Authors:** Alema Puzovic, Massimiliano Rinaldi, Maja Mikulic‐Petkovsek

**Affiliations:** ^1^ Biotechnical Faculty, Agronomy Department University of Ljubljana Ljubljana Slovenia; ^2^ Department of Food and Drug University of Parma Parma Italy

**Keywords:** anthocyanins, berry pomace, bioactive compounds, electroporation, innovative technologies, process optimization

## Abstract

This study investigated the effect of pulsed electric field (PEF) treatment on cell disintegration and phenolic extractability from haskap (*Lonicera caerulea* L.) pomace generated after juice processing. Pomace samples were subjected to 16 PEF conditions combining electric field strengths of 1.0 and 1.5 kV/cm, pulse frequencies of 5 and 10 Hz, and specific energy ranging from 1 to 12.3 kJ/kg. Cell membrane permeabilization was quantified using the cell disintegration index (*Z*_p_), whereas phenolic compounds were profiled by HPLC–PDA–mass spectrometer (MS). One‐way analysis of variance (ANOVA) revealed significant differences among treatments for *Z*_p_ and all phenolic groups. Moderate to high‐intensity treatments (1.5 kV/cm, 5–10 Hz, 5–12.3 kJ/kg) produced the highest *Z*_p_ values (0.10–0.12) and resulted in the greatest enhancement of phenolic extraction, particularly anthocyanins, flavonols, flavanols, and hydroxycinnamic acids. Anthocyanins exhibited the strongest response, with up to a 2.6‐fold increase compared to the untreated control. Multivariate and regression analyses identified electric field strength and pulse frequency as the main factors influencing cell disintegration and phenolic extraction. In contrast, specific energy showed mainly nonsignificant or negative regression coefficients, suggesting it was not a relevant predictor of phenolic extractability under the tested conditions. These findings provide quantitative insight into PEF process optimization for enhanced phenolic extraction from haskap berry pomace.

## 1 Introduction

Fruits and vegetables play a central role in maintaining human health, with dark‐colored berries particularly valued for their nutritional profile and rich phytochemical content (Cheng et al. 2023). Over the past two decades, the global cultivation and market demand for berries have expanded rapidly, especially because of their ornamental, functional, and edible properties (Cheng et al. 2023). Among these, haskap (*Lonicera caerulea* L.), also known as honeyberry or honeysuckle, has emerged as a promising northern berry species. Traditionally consumed in Russia, Japan, and Northeastern China, haskap is recognized for its therapeutic properties. As an excellent source of bioactive compounds, scientific interest in this species has increased in recent years (Cheng et al. 2023; Wang et al. 2023).

Haskap berries are known for their high contents of phenolic compounds, anthocyanins, organic acids, vitamin C, and minerals, which together contribute to their strong antioxidant capacity (Česonienė et al. 2021). In addition to these compounds, haskap berries contain free sugars, terpenoids, iridoids, and carotenoids, with cyanidin‐3‐*O*‐glucoside identified as the predominant anthocyanin responsible for the characteristic dark–purple color and antioxidant activity of the fruit (Cheng et al. 2023). These bioactive compounds, including chlorogenic acid, ferulic acid and its derivatives, and rutin, contribute to a wide range of physiological effects, such as anticancer, anti‐inflammatory, hepatoprotective, antidiabetic, anti‐obesity, and skin‐protective properties (Cheng et al. 2023; Zehfus et al. 2021). Numerous studies have also reported the potential role of haskap polyphenols in regulating oxidative stress, modulating inflammatory pathways, and protecting against cellular damage, additionally supporting the classification of haskap as a functional food with promising nutraceutical applications (Cheng et al. 2023). Haskap has also been reported to contain higher levels of ascorbic acid and anthocyanins than other commercially recognized “super berries,” including blueberries (Wang et al. 2025). Beyond food applications, haskap‐derived bioactive compounds have also attracted interest for use in cosmetics, intelligent packaging films, dietary supplements, and functional beverages because of their antioxidant, coloring, and antimicrobial properties (Cheng et al. 2023). However, like many highly perishable berries, haskap fruit is prone to moisture loss, shriveling, skin rupture, juice leakage, and mold growth, resulting in a very short shelf life and substantial postharvest losses (Wang et al. 2025). Therefore, despite its considerable nutritional potential, the commercial value of fresh haskap remains underdeveloped, and it increases the importance of processing to stabilize its quality and extend its utilization. Even though haskap has increasingly been incorporated into value‐added products, such as juices, wines, jams, dried fruits, and nutraceuticals (Cheng et al. 2023), research on haskap processing remains sparse, particularly regarding the retention or recovery of its valuable phenolic compounds from by‐products. Juice extraction generates substantial quantities of pomace, including skins, seeds, and pulp residues, that still contain high levels of bioactive compounds, which is an environmental and economic challenge due to its limited high‐value applications (Pedro et al. 2024). Despite the potential of utilization of fruit by‐products as sources of natural additives, functional ingredients, and bioactives for foods, cosmetics, and pharmaceutical applications, the extraction of intracellular compounds from berry pomace can be challenging because of the structural resistance of plant envelopes that can limit mass transfer and reduce extraction efficiency in conventional solid–liquid extraction processes (Rrucaj et al. 2023; Carpentieri, Ferrari, et al. 2022). This has increased interest in nonthermal cell‐disruption technologies that can weaken or perforate cell membranes, thereby enhancing the release of phenolics while reducing solvent, energy, and processing time requirements (Carpentieri, Ferrari, et al. 2022). Among these technologies, pulsed electric field (PEF) treatment has attracted significant attention as an innovative and nonthermal technique for improving mass transfer in plant tissues (Carpentieri, Režek Jambrak, et al. 2022). PEF involves the application of short electrical pulses that induce electroporation, that is, an increase in cell membrane permeability through pore formation (Carpentieri, Ferrari, et al. 2022; Raso et al. 2016). This phenomenon enhances the diffusion of intracellular compounds and has been widely explored for microbial inactivation, extraction intensification, and improved dehydration efficiency of fruits and vegetables (Rrucaj et al. 2023; Raso et al. 2016).

Previous studies on grape, blueberry, and sweet and sour cherry pomace have shown that PEF can substantially increase the extraction of polyphenols and anthocyanins, even at moderate electric field strengths and low specific energy inputs (Bobinaitė et al. 2015; Carpentieri, Ferrari, et al. 2022; Pataro et al. 2017; Rrucaj et al. 2023). Increases of more than 12% in total polyphenol yield and up to 17% in anthocyanin concentration have been reported for grape skins following PEF pretreatment, implying the potential of PEF to valorize berry processing residues by improving extraction efficiency while maintaining the integrity of thermolabile compounds (Corrales et al. 2008). Nonthermal extraction technologies, including ultrasound, microwave, enzyme, and supercritical fluid‐assisted extraction, have increasingly been investigated to improve the recovery of bioactive compounds from plant materials (Zahra et al. 2025). However, despite their advantages over conventional extraction approaches, these methods may still involve intensive solvent use, prolonged processing times, partial degradation of thermosensitive compounds, or limitations regarding industrial implementation (Ranjha et al. 2021). Among emerging technologies, PEF treatment has attracted considerable interest as a sustainable nonthermal approach because it increases cell membrane permeability through electroporation, thereby facilitating mass transfer and the release of intracellular compounds (Carpentieri, Ferrari, et al. 2022; Ranjha et al. 2021). As PEF treatments are typically performed under mild thermal conditions and short treatment durations, they may improve the recovery of thermolabile phenolic compounds while better preserving their functional properties (Alkanan et al. 2024). Furthermore, PEF technology has been recognized for its potential integration into continuous industrial processing systems with reduced solvent and energy requirements compared to conventional extraction techniques (Alkanan et al. 2024; Ranjha et al. 2021). In the context of fruit‐processing by‐products, these characteristics are particularly relevant because PEF may be incorporated upstream of extraction processes as a pretreatment step for large volumes of pomace streams, potentially improving recovery efficiency while supporting sustainable and circular processing strategies (Carpentieri et al. 2023; Rrucaj et al. 2024).

Although PEF‐assisted extraction of phenolic compounds from berry matrices has been previously reported, available studies have predominantly focused on intact fruit tissues or conventional berry pomaces. However, haskap pomace represents an underexplored by‐product characterized by thick berry skins, residual pulp, seeds, and a high proportion of insoluble fiber fractions, which may alter electroporation behavior and mass‐transfer mechanisms compared with intact tissues (Jurevičiūtė et al. 2022). Therefore, understanding the relationship between PEF‐induced cell disintegration and phenolic extractability in haskap pomace may provide insight into extraction behavior and improve understanding of PEF factors affecting phenolic recovery in this matrix. To the best of our knowledge, no study has specifically investigated the application of PEF technology to haskap berry pomace.

Previous studies have indicated that haskap by‐products still retain significant concentrations of phenolic acids, anthocyanins, and minerals after processing, indicating their strong potential for valorization as sources of functional ingredients and natural antioxidants (Cheng et al. 2023). Therefore, given the exceptionally high phenolic content of haskap pomace and the growing interest in sustainable processing and by‐product utilization, further research is required to determine the extent to which PEF can enhance the recovery of bioactive compounds from this material.

Therefore, the aim of this study is to evaluate the extraction of phenolic compounds from the haskap pomace and to examine the extent of cell disintegration induced by PEF treatment after juice processing, with the goal of improving the valorization of haskap by‐products.

## 2 Materials and Methods

### 2.1 Pomace Production and PEF Treatments

Fresh haskap pomace (*L. caerulea* L. cv. “Aurora”) was obtained as a by‐product of juice extraction. Whole berries were pressed using a Hydro PARA‐Press (Paul Arauner GmbH, Germany) at 3.5 bar for 15 min. Juice extraction of 2.1 kg fresh berries generated approximately 660 g of pomace, corresponding to a pomace yield of ca. 32% on a fresh weight basis. The resulting pomace was collected immediately after pressing and divided into three fractions: fresh pomace, frozen/thawed pomace, and pomace intended for PEF treatment.

For PEF processing, a batch‐mode EPULSUS‐LBM1B‐15 system (Energy Pulse Systems, Lisbon, Portugal), equipped with a parallel stainless‐steel plate treatment chamber (10 cm electrode gap), was used. For each treatment, 50 g of pomace was placed in the chamber and mixed with 150 mL of tap water to achieve a solid–liquid ratio of 1:3, which was necessary to ensure adequate electrical conductivity and to enable uniform electric field distribution across the pomace matrix during treatment. Voltage and current waveforms were monitored using a PicoScope 2000 Series mixed‐signal oscilloscope (Pico Technology, UK) connected to the PEF system, and signals were recorded using PicoScope 7 T&M software.

The following treatments were applied:

- Control (untreated pomace).
- Freezing/thawing treatment—prior to analysis, pomace was frozen at −18°C and thawed overnight at 4°C (approximately 12–14 h).
- PEF treatments—PEF treatments were conducted using monopolar square‐wave pulses at an electric field strength of either 1.0 or 1.5 kV/cm, pulse durations of ±10 µs, and pulse frequencies of 5 or 10 Hz, depending on the treatment. Sixteen PEF conditions were tested, and all of the applied parameters are presented in Table 1. Temperature and electrical conductivity were measured before and after treatment using a portable conductivity meter (mod. 913, Knick Elektronische, Berlin, Germany).

**TABLE 1:** Processing parameters applied to haskap pomace.

Treatment	Δ*T* (°C)	*E* (kV/cm)	*τ* (±µs)	*f* (Hz)	Number of pulses	*W* (kJ/kg)
**Control**	na	—	—	—	—	—
**Frozen**	na	—	—	—	—	—
**PEF 1**	1.25 ± 0.63	1	10	5	140	1
**PEF 2**	1 ± 0.58	1	10	5	720	5
**PEF 3**	6 ± 0.71	1	10	5	1430	10.3
**PEF 4**	6.38 ± 0.24	1	10	5	1750	12.3
**PEF 5**	3.25 ± 0.63	1.5	10	5	30	1
**PEF 6**	0.75 ± 0.25	1.5	10	5	150	5
**PEF 7**	6.0 ± 0.1	1.5	10	5	300	10.3
**PEF 8**	7.63 ± 0.63	1.5	10	5	370	12.3
**PEF 9**	4.75 ± 0.25	1	10	10	70	1
**PEF 10**	0.75 ± 0.25	1	10	10	380	5
**PEF 11**	5.0 ± 0.41	1	10	10	730	10.3
**PEF 12**	8.38 ± 0.47	1	10	10	890	12.3
**PEF 13**	1.53 ± 0.38	1.5	10	10	15	1
**PEF 14**	6.83 ± 1.12	1.5	10	10	75	5
**PEF 15**	8.75 ± 0.48	1.5	10	10	150	10.3
**PEF 16**	9.0 ± 0.71	1.5	10	10	185	12.3

*Note*: Δ*T* values are reported as mean ± SE (*n *= 4). na, refers to not applicable. Frozen samples were stored at −18°C and used as a fully permeabilized reference for *Z*_p_ calculation; temperature rise during treatment was not applicable.

Abbreviation: PEF, pulsed electric field.

Specific energy input (*W*) was considered an integrated descriptor of treatment intensity and was determined according to established PEF reporting recommendations (Raso et al. 2016). As specific energy is derived from multiple treatment variables, including electric field strength, pulse duration, pulse number, and the characteristics of the treated material, it does not represent an independent experimental factor. In the present study, pulse duration was kept constant (10 µs), whereas electric field strength (1.0 and 1.5 kV/cm) and pulse frequency (5 and 10 Hz) were varied independently. The number of pulses was adjusted to achieve predefined specific energy levels (1, 5, 10.3, and 12.3 kJ/kg). This approach enabled the assessment of the relative influence of individual PEF parameters on cell disintegration and phenolic extractability to be evaluated independently from the total energy input. Therefore, by varying electric field strength and pulse frequency independently while maintaining similar energy inputs across selected treatments, the study aimed to clarify whether cell disintegration and phenolic extraction were primarily affected by total delivered energy or by distinct electroporation‐related parameters. The complete experimental matrix and replicate structure are presented in Table S1.

### 2.2 Cell Disintegration Index

Considering that PEF treatment causes electroporation, the cell disintegration/permeabilization index (*Z*) was calculated to measure the possible cell degradation/damage induced by PEF, according to the formula of (Lebovka et al. 2002)

Z=σ−σu/σd−σu

where *σ* is the measured electrical conductivity of the pomace sample, and the subscripts *u* and *d* refer to the conductivities of untreated and frozen/thawed samples, respectively. Electrical impedance measurements were performed using an LCR bridge (CR400, Thurlby Thandar Instruments, UK) at test frequencies of 1 and 10 kHz. The permeabilization index can be defined as the fraction of completely electroporated cells in the food tissue and can be used as response variable to identify the optimal PEF treatment conditions (Carpentieri, Ferrari, et al. 2022; Lebovka and Vorobiev 2017).

### 2.3 Extraction and Quantification of Phenolic Compounds

Phenolic compounds were extracted and analyzed following the procedure described by Mikulic‐Petkovsek et al. (2020), with minor modifications. Approximately 5.1–5.3 g of pomace was mixed with 5 mL of 80% aqueous methanol (v/v) and extracted for 30 min in an ultrasonic water bath (Langford Sonomatic 575, 40 kHz, 300 W) under cooled conditions (approximately 4°C), with ice added periodically to maintain a low extraction temperature. Following the extraction, samples were centrifuged using an Eppendorf 5810 R centrifuge (Eppendorf, Germany) at 6000 rpm for 5 min at 4°C and filtered through 0.2 µm cellulose filters (Macherey‐Nagel, Germany). Phenolic profiling was performed using an HPLC system (Finnigan Surveyor, Thermo Fisher Scientific, San Jose, CA, USA) equipped with a photodiode array detector operating at 280, 350, and 530 nm, coupled to a mass spectrometer (MS) for compound identification. Separation was achieved on a Gemini C18 column (Phenomenex, Torrance, CA, USA) maintained at 25°C.

The mobile phases consisted of bidistilled water/acetonitrile/formic acid (96.9/3.0/0.1, v/v/v) as mobile phase A and acetonitrile/bidistilled water/formic acid (96.9/3.0/0.1, v/v/v) as mobile phase B. The elution gradient started at 5% B and increased to 20% B over the first 15 min, followed by a linear increase to 30% B in the next 5 min, an isocratic step of 5 min, a further increase to 90% B over 5 min, and a final 15 min isocratic phase before re‐equilibration to initial conditions. Anthocyanins were detected in positive ionization mode, whereas all other phenolic compounds were analyzed in negative ionization mode using full‐scan‐data‐dependent MS*^n^* acquisition over *m*/*z* 115–1900. MS parameters included a capillary temperature of 250°C, sheath and auxiliary gas flows of 60 and 15 U, respectively, a source voltage of 3 kV, and normalized collision energy of 20%–35%.

Data acquisition and processing were performed using Xcalibur 4.7 software (Thermo Scientific, Waltham, MA, USA). Phenolic compounds were identified by comparing retention times, PDA spectra, molecular ions, and MS*^n^* fragmentation patterns with authentic standards and previously published literature data to minimize false‐positive identifications. Quantification was conducted using external calibration curves generated from standards. The identified compounds mainly included anthocyanins (cyanidin, delphinidin, pelargonidin, and peonidin‐derived glycosides), flavones (genistein and luteolin glycosides), flavanones (naringenin hexoside), flavonols (quercetin and isorhamnetin glycosides), flavanols (catechin and procyanidin oligomers), hydroxybenzoic acids (ellagic acid hexoside), and hydroxycinnamic acids (HCAs) (caffeic, dicaffeoylquinic, *p*‐coumaric, and feruloylquinic acid derivatives). Phenolic concentrations were expressed in mg/kg.

### 2.4 Statistical Analysis

Statistical analysis was performed using the R‐commander statistical software version 4.3.0 (R Formation for Statistical Computing, Auckland, New Zealand). Differences between the treatments were evaluated using one‐way analysis of variance (ANOVA), followed by Tukey's post hoc test for multiple comparisons at a 95% confidence level. All measurements were performed as four independent experimental (process) replicates (*n* = 4), each consisting of a separate 50 g batch of fresh haskap pomace independently subjected to PEF treatment and extraction. Results are presented as mean values ± standard error (mean ± SE).

In addition to univariate analysis, multiple linear regression models were used to investigate the influence of PEF parameters (*E*, *f*, and *W*) on phenolic compound concentrations. Regression diagnostics and coefficient extraction were performed in R using the built‐in modeling functions within R‐commander.

Multivariate analysis was conducted using principal component analysis (PCA) with the FactoMineR package in R (version 4.3.0), and visualizations were created using factoextra package.

## 3 Results and Discussion

### 3.1 Cell Disintegration Index

**FIGURE 1:**
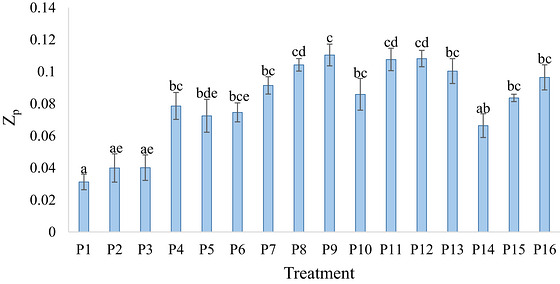
Cell disintegration index (*Z*_p_) measured in PEF‐treated haskap pomace. The values are the means of four repetitions. Mean values followed by a different letter are significantly different according to Tukey's test (*p* ≤ 0.05).

Measurements of the changes in the electrophysical properties, such as complex electrical impedance of untreated and PEF‐treated biological materials, have been suggested as a simple and reliable method to obtain a measurement of the extent of damaged cells (Donsì et al. 2010; Pataro et al. 2011). Therefore, the cell disintegration index (*Z*_p_) provided a quantitative measure of electroporation efficiency in haskap pomace and showed differences in membrane disruption across the applied PEF conditions. One‐way ANOVA confirmed that *Z*_p_ values differed significantly among treatments (*p *< 0.001), indicating a strong dependence of cell disintegration on PEF intensity (Figure 1). It has been demonstrated that *Z*_p_ is a reliable indicator of the degree of cell membrane permeabilization induced by PEF treatment in diverse agri‐food by‐product tissues, including those derived from winemaking processes (Carpentieri et al. 2023).

In the present study, low‐intensity treatments (P1–P3), conducted at 1 kV/cm and low specific energy inputs (1–5 kJ/kg), produced the lowest *Z*_p_ values (0.03–0.04). These treatments formed the lowest statistical group (a‐ae) and did not differ significantly from each other (*p *> 0.05). These values are consistent with reversible electroporation, as electroporation can result in reversible or irreversible effects, and reversible membrane permeabilization is typically induced by low‐intensity PEF treatment (Vaessen et al. 2019). Under these conditions, the induced transmembrane potential may have been insufficient to overcome the structural rigidity of haskap berry skins, similar to the findings of Lamanauskas et al. (2015), where low treatments were too mild to induce further permeabilization of blueberry tissue. Haskap pomace exhibits a mechanically resistant matrix comparable to red grape pomace, where the higher resistance to the electropermeabilization treatment might be ascribed to their higher content in insoluble dietary fibers (cellulose, hemicellulose, and lignin) (Carpentieri et al. 2023), which statistically explains the consistently low *Z*_p_ values observed for P1–P3.

Moderate‐intensity treatments (P4–P7; 1–1.5 kV/cm, 5–12.3 kJ/kg) produced intermediate *Z*_p_ values (0.06–0.09). These treatments were statistically distinct from the low‐intensity group (*p* < 0.05) with most values belonging to the b‐bc significance groups, which aligns with previous findings showing that the permeabilization degree depended mainly on the applied electric field rather than on the energy input, and that increasing the field strength from 1 to 5 kV/cm, the *Z*_p_ values significantly increased (Boussetta et al. 2009; Lamanauskas et al. 2015; Pataro et al. 2019). Similarly, in the study from Carpentieri, Ferrari, et al. (2022), the electric field strength was the factor that mostly affected the observed response, whereas the effects of energy input become more evident only at lower field strengths.

The highest *Z*_p_ values (0.10–0.12) were observed for treatments P8–P13, which included higher electric field strength (1.5 kV/cm) with both pulse frequencies (5 and 10 Hz) and moderate‐to‐high‐energy inputs (5–12.3 kJ/kg). These treatments were assigned to the highest significance groups (c‐cd) and possibly caused irreversible electroporation, consistent with the principle that high‐intensity fields disrupt membrane integrity, considering that the application of high‐voltage impulses results in the disruption of the cell membrane, rendering it permeable to small molecules (Raso et al. 2016). In line with prior studies, increases in *Z*_p_ indicate improvements in mass transfer because permeabilization of the cell membrane enhanced mass transfer through the membrane, resulting in improved extraction of various biochemical compounds (Frontuto et al. 2019; Maskooki and Eshtiaghi 2011).

Interestingly, P14–P16 treatments were classified within lower statistical groupings (ab‐bc), indicating that *Z*_p_ values declined. These PEF treatments combined high field strength (1.5 kV/cm), high pulse frequency (10 Hz), and the highest specific energy inputs (10.3–12.3 kJ/kg). Higher pulse frequencies and elevated specific energy inputs may induce electroporation saturation phenomena, including dielectric relaxation and accelerated pore resealing, thereby counteracting further membrane permeabilization. This has been reported in various plant tissues, including grape and raspberry by‐products and orange skins, and where further increases in energy input did not enhance *Z*_p_ and permeabilization tended to level off despite more intense treatments (Boussetta et al. 2009; Lamanauskas et al. 2016; Luengo et al. 2013). Moreover, the electroporation response depends on frequency and is strongly influenced by tissue electrical properties, confirming that permeabilization and mass transfer are governed by complex interactions between PEF parameters and material characteristics (Lamanauskas et al. 2015, 2016).

Reported *Z*_p_ values for plant tissues vary considerably depending on the structural characteristics of the material and treatment conditions. Previous studies reported ranges of 0.2–0.9 for sugar beet (Maskooki and Eshtiaghi 2011), 0.1–0.3 for orange peel (Luengo et al. 2013), and 0–0.8 for potato tissue (Lebovka et al. 2014). Compared with these matrices, the *Z*_p_ values observed for haskap pomace (0.03–0.12) were relatively low. However, studies on different plant materials have demonstrated that cell disintegration responses vary substantially according to tissue structure, cell geometry, porosity, and composition (Alam et al. 2018; Shorstkii et al. 2022). Therefore, direct comparison should be interpreted cautiously because electroporation behavior is strongly matrix dependent. Additionally, unlike intact tissues commonly used in PEF studies, haskap pomace is a heterogeneous by‐product consisting primarily of disrupted skins, residual pulp, and seeds generated during juice extraction (Jurevičiūtė et al. 2022). Berry pomaces are known to contain high levels of insoluble dietary fiber, including cellulose, hemicellulose, and lignin‐rich fractions, which may contribute to elevated water‐holding capacity and dense structural organization (Jurevičiūtė et al. 2022). Such components may potentially reduce tissue porosity and limit electric field efficiency through the matrix, thereby decreasing the efficiency of membrane permeabilization.

Additionally, previous studies have shown that increasing electric field strength, pulse number, or specific energy generally increases cell disintegration only up to a saturation region, beyond which further increases produce limited effects (Dastangoo et al. 2020; Llavata et al. 2024; Shorstkii et al. 2022). The relatively moderate electric field strengths applied in the present study (1–1.5 kV/cm) may, therefore, have induced only partial membrane permeabilization in haskap pomace. Consequently, the low *Z*_p_ values observed do not necessarily indicate ineffective treatment but rather suggest that electroporation efficiency in haskap pomace may be constrained by matrix‐specific structural properties and, therefore, requires parameter optimization.

### 3.2 Extractability of Phenolic Compounds

**FIGURE 2:**
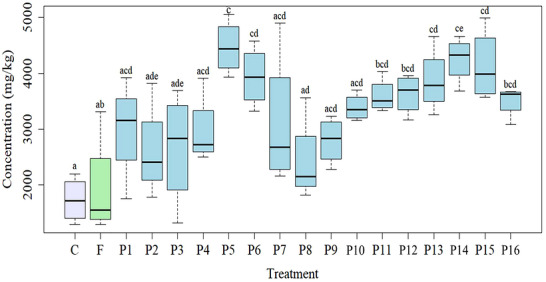
Concentration of total phenolics expressed as mg/kg in haskap pomace samples. The values are the means of four repetitions. Mean values followed by a different letter are significantly different according to Tukey's test (*p* ≤ 0.05).

The concentration and composition of phenolic compounds extracted from haskap pomace are presented in Figure 2, Table A1, and Tables S2–S9. Figure 2 summarizes the concentration of total phenolics quantified in haskap pomace samples across the applied treatments and control. As presented, concentration of total phenolics ranged from approximately 1293 mg/kg in untreated control samples to 5059 mg/kg in P5‐treated samples, corresponding to an almost 3.9‐fold increase. Significant differences among treatments were observed according to Tukey's test (*p* ≤ 0.05), indicating that PEF conditions significantly affected the recovery of phenolic compounds. The untreated control exhibited the lowest total phenolic concentration and belonged only to significance group a, whereas several PEF‐treated samples, particularly P5, P14, and P15, appeared among the treatments associated with the highest significance categories (c and/or d). However, these treatments also shared significance letters with several intermediate treatments, namely, P4, P6–P13, and P16, implying that multiple treatment conditions produced comparable phenolic recovery.

Furthermore, a total of 86 individual phenolic compounds belonging to seven phenolic groups were identified, including anthocyanins (10 compounds), flavanols (16), flavonols (25), flavones (5), flavanones (4), HCAs (15), hydroxybenzoic acids (1), and iridoids (1). Among the total phenolics, anthocyanins represented the predominant fraction, accounting for approximately 68%–87% of total phenolics depending on the treatment. Flavanols were the second most abundant group, contributing approximately 4%–9%, whereas HCAs accounted for approximately 5%–7% of total phenolics. Flavonols represented around 1.5%–2.5%, whereas iridoids contributed less than 1.1%. Flavones, flavanones, and hydroxybenzoic acids were detected only in trace amounts and together represented less than 1% of total phenolics. This compositional profile is in agreement with previous studies describing haskap berries as anthocyanin‐rich fruits in which cyanidin derivatives largely determine antioxidant potential and biological activity (Cheng et al. 2023). As presented in Table A1, PEF treatment significantly enhanced the extractability of most phenolic groups, and these improvements followed the pattern observed for the *Z*_p_ results. Treatments associated with higher *Z*_p_ values, particularly P5–P12, generally resulted in the greatest increases in phenolic concentrations compared with the untreated control and lower intensity treatments. Similar observations have been reported previously, where enhanced extraction efficiency during PEF‐assisted extraction was attributed to membrane permeabilization and increased diffusivity of intracellular compounds (Lončarić et al. 2020).

Concentration of total anthocyanins increased significantly (*p* < 0.001) from 1437.50 mg/kg in the untreated control to the highest concentration observed in P5 (3715.08 mg/kg), corresponding to a 2.6‐fold increase relative to the control. As presented, treatments P5, P6, and P13–P15 showed significantly higher anthocyanin concentrations. The strong anthocyanin response can be explained by their intracellular localization and chemistry. Anthocyanins are predominantly stored in vacuoles of epidermal and subepidermal tissues and are therefore highly susceptible to release following membrane permeabilization (Sharma et al. 2024). Their characteristic of a high‐water solubility further facilitates diffusion once cellular barriers are disrupted. Previous studies similarly reported that increasing electric field intensity enhances anthocyanin extraction because greater transmembrane potential induces membrane rupture and promotes intracellular release (Zhou et al. 2015).

To better explain treatment effects, individual compounds were evaluated in addition to phenolic groups. Anthocyanins consisted mainly of cyanidin derivatives, including cyanidin‐3‐*O*‐glucoside, cyanidin‐3‐*O*‐rutinoside, and cyanidin‐3,5‐diglucoside together with peonidin and pelargonidin derivatives. Cyanidin compounds represented the dominant individual anthocyanins. Among these, cyanidin‐3‐*O*‐glucoside was the major compound across all treatments and showed significant differences among samples (*p* ≤ 0.05), increasing from 1127.75 mg/kg in control samples to approximately 2500–3150 mg/kg in P13–P16 treatments. Cyanidin‐3‐*O*‐rutinoside exhibited a similar trend and increased significantly from 23.98 mg/kg in control samples to approximately 65–82 mg/kg under P10–P14 treatments. Cyanidin‐3,5‐diglucoside also increased significantly in P13–P15 treatments, whereas cyanidin‐3‐*O*‐rhamnosyl‐hexoside remained relatively stable. These statistical differences indicate compound‐specific extraction behavior. Although anthocyanins generally increased under moderate and high electroporation conditions, overlapping Tukey groupings among some treatments suggest that the magnitude of improvement depended on treatment intensity and molecular structure. Such differences may reflect variability in polarity, glycosylation pattern, and interactions with surrounding cellular structures (Ikhsan et al. 2026; Xue et al. 2024).

PEF treatment also significantly enhanced flavonol and flavanol concentrations in comparison to control samples (*p* = 0.003 and *p* < 0.001, respectively). As presented in Table A1, concentration of flavonols increased from 36.40 mg/kg in control samples to values exceeding 80 mg/kg in treatments P5 and P12, with the highest average concentration observed in P14 (85.66 mg/kg). Flavanols exhibited an even better response, increasing from 116.36 mg/kg in control samples to 401.97 mg/kg in P15, representing a 3.5‐fold increase. Additionally, flavanols were the second most abundant phenolic group and consisted primarily of procyanidin oligomers together with epicatechin and catechin. Among the individual compounds, procyanidin dimer 1 and procyanidin dimer 3 showed the highest concentrations and response to PEF treatment. Procyanidin dimer 1 increased significantly from 21.59 mg/kg in control samples to 114.44 mg/kg in P15, whereas procyanidin dimer 3 increased from 26.94 to 118.12 mg/kg. Similar trends were observed for dimers 2 and 4, whereas epicatechin increased from 14.01 to 35.73 mg/kg. In contrast, catechin showed comparatively lower concentrations and weaker treatment responses. These results, specifically regarding the procyanidin oligomers, may imply differences in localization and binding interactions. As procyanidins are commonly associated with berry skins and cell wall structures, they may become more extractable following disruption of structural barriers (Valencia‐Hernandez et al. 2021).

Among non‐flavonoid phenolics, HCA and iridoids also exhibited significant increases across treatments (*p *= 0.003 and *p *< 0.001, respectively). HCAs were dominated by chlorogenic acid derivatives, particularly 3‐*O*‐caffeoylquinic acid and 5‐*O*‐caffeoylquinic acid, which represented the major HCA fractions in all samples. Concentration of 3‐*O*‐caffeoylquinic acid increased from 54.67 mg/kg in control samples to 141.23 mg/kg in P5 and showed significantly higher concentrations in several PEF treatments. Similarly, 5‐*O*‐caffeoylquinic acid increased from 61.19 to 124.49 mg/kg. However, overlapping significance groups indicated that treatment effects were less pronounced than observed for anthocyanins and flavanols.

The more moderate statistical response of HCA compounds may be explained by differences in intracellular distribution and matrix interactions. Unlike anthocyanins, chlorogenic acid derivatives may be distributed across multiple cellular compartments and can additionally associate with structural components of plant tissues, limiting release efficiency (Baker et al. 2017; Moreira et al. 2018). Overall, on the basis of the results, increased *Z*_p_ values were generally associated with improved extraction of phenolic compounds; however, the response was also dependent on the phenolic compounds. This suggests that, besides the intensity of the treatment, the extractability of phenolics was also affected by their chemical characteristics and tissue distribution, including molecular structure, polarity, intracellular localization, and affinity for structural components of the plant material (Travis et al. 2026).

### 3.3 Multiple Regression Analysis

Multiple regression modeling showed that the three tested PEF parameters had different effects on phenolic extractability from haskap pomace, as summarized in Table A2. Across nearly all phenolic groups, electric field strength was the dominant predictor, with strong and significant positive coefficients for anthocyanins, total phenolics, flavanols, flavones, HCA, and iridoids. This is in agreement with previous studies from Boussetta et al. (2009) and Carpentieri et al. (2023) showing that the permeabilization degree shows a great dependence on the electric field strength, and that the electric field strength was the factor that mostly affected the observed response. Consistent with this, earlier studies reported that increasing the field strength from 1 to 5 kV/cm significantly increased *Z*_p_, and that the electric field strength applied showed a positive influence on the *Z*_p_ value (Boussetta et al. 2009; Carpentieri et al. 2023). Pulse frequency also showed significant positive effects for several phenolic groups, including anthocyanins, total phenolics, iridoids, flavanones, and flavones. Previous impedance analyses provide further evidence that electroporation responses in plant matrices are affected by pulse frequency, as stated in the study by Lamanauskas et al. (2015), which found that the phase angle shift was most pronounced at medium frequencies. However, some compounds, namely, HBA and certain flavonols, showed no significant frequency response. Considering that HBAs have a higher number of hydroxyl groups, it has been reported by Kutraite et al. (2025) that their extraction efficiency from plant materials can be restricted; hence, these results could be caused by structural or localization constraints within the haskap tissue matrix.

In contrast, specific energy showed predominantly negative or nonsignificant regression coefficients across all phenolic groups. This reflects the *Z*_p_ results where high‐energy treatments (P14–P16) resulted in lower cell disintegration despite delivering more total energy. Similarly, Boussetta et al. (2009) and Lamanauskas et al. (2016) reported that further increases in total specific energy did not enhance cell permeabilization in grape and raspberry by‐products, suggesting that excessive energy may cause localized heating, dielectric relaxation, or membrane resealing, which counteract extraction rather than enhancing it.

Treatments applied at 1.5 kV/cm consistently resulted in the highest cell disintegration (*Z*_p_ = 0.10–0.12), particularly when combined with pulse frequencies of 5–10 Hz and moderate specific energy inputs (5–12.3 kJ/kg), and these conditions were associated with the highest concentrations of most phenolic groups. In contrast, treatments with *E* = 1 kV/cm and *W* = 1–5 kJ/kg produced only limited membrane permeabilization (*Z*_p_ = 0.03–0.04) and correspondingly low phenolic extractability, whereas excessively high‐energy inputs (10.3–12.3 kJ/kg at 10 Hz) led to a reduction in *Z*_p_, indicating a possible electroporation saturation or counteracting effects.

### 3.4 PCA Structure and Variable Contributions

**FIGURE 3:**
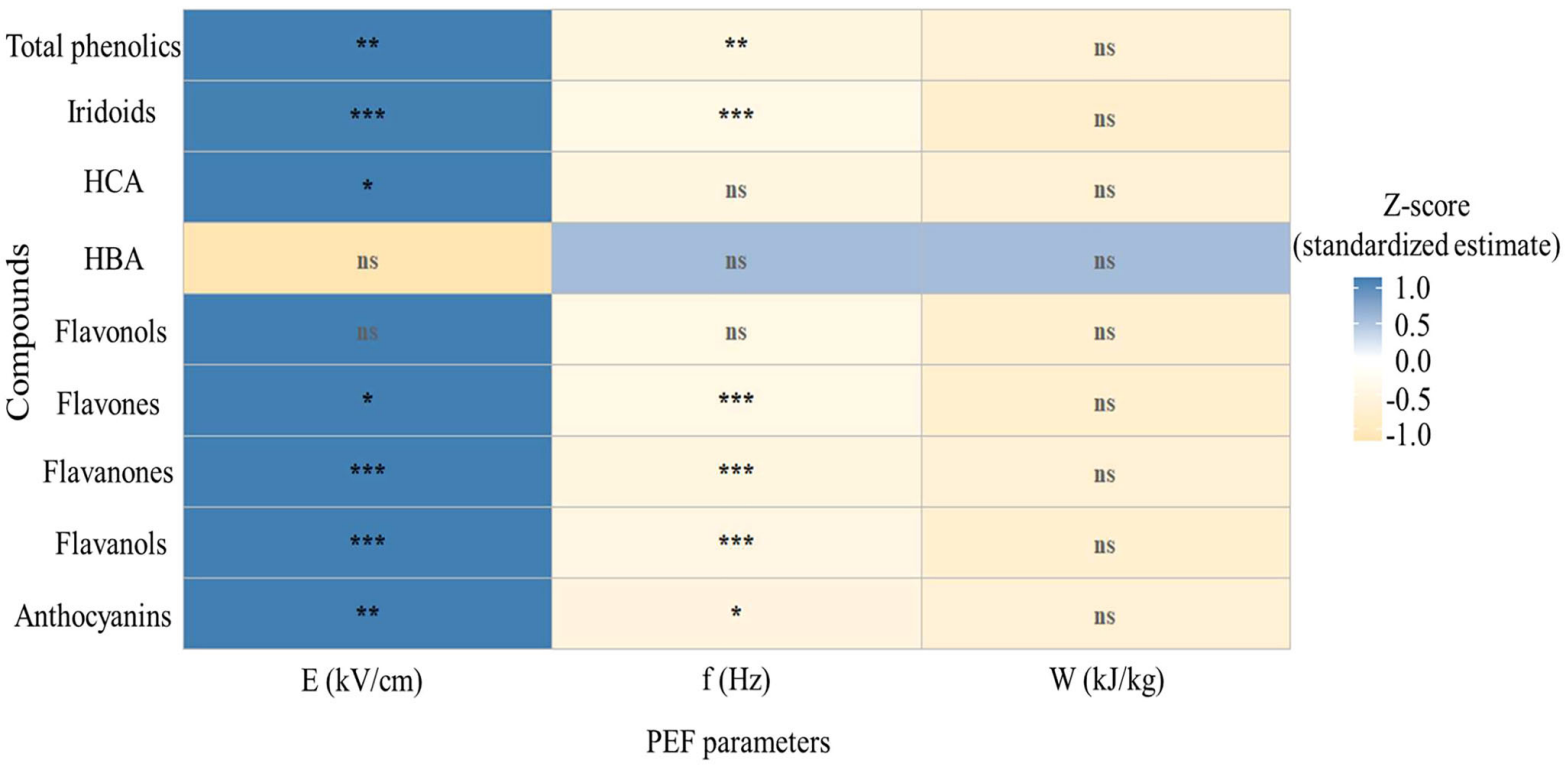
Standardized regression coefficients for PEF process parameters affecting phenolic extractability. HCA, hydroxycinnamic acid; PEF, pulsed electric field.

**FIGURE 4:**
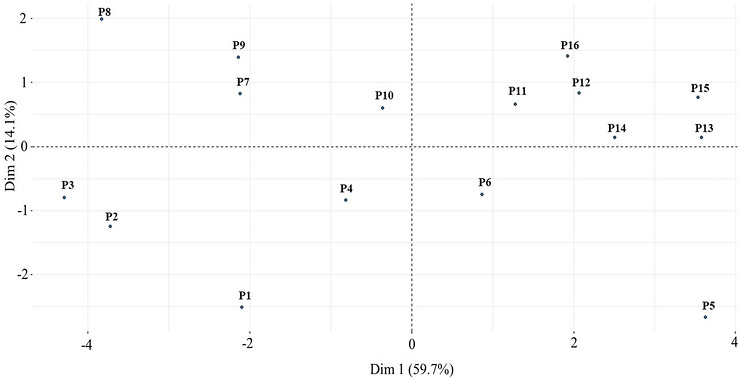
PCA score plot of samples.

The PCA provided a comprehensive overview of the relationships among PEF parameters, cell disintegration (*Z*_p_), and phenolic extractability (Figures 3 and 4). The first principal component (Dim‐1), explaining 59.7% of the variance, clearly separated treatments according to electric field strength and pulse frequency. High‐intensity treatments (P5, P13–P16) were positioned on the positive side of Dim‐1, whereas low‐intensity treatments (P1–P3) clustered negatively, representing varying degrees of electroporation efficiency. This pattern is consistent with previous studies indicating that electric field strength was the main factor affecting the observed response, and that increasing field strength substantially enhances membrane permeabilization, with *Z*_p_ rising significantly between 1 and 5 kV/cm (Lamanauskas et al. 2015).

The alignment of E and frequency vectors with *Z*_p_ and most phenolic groups in the correlation plot confirms that treatments causing greater membrane disruption also produced higher phenolic release. This agrees with literature showing that enhanced extraction results directly from electroporation, as PEF treatment facilitates solvent penetration into the cytoplasm and the mass transfer of solubilized intracellular compounds (Rrucaj et al. 2024). The strong co‐orientation of anthocyanins, flavonols, flavanols, flavones, flavanones, iridoids, and HCA along Dim‐1 further supports the observation that most phenolic classes respond similarly to electroporation conditions, consistent with reports that PEF treatments can increase polyphenol recovery by up to 205% in fruit by‐products (Chatzimitakos et al. 2023). In contrast, specific energy input (*W*) pointed in the opposite direction along Dim‐1, indicating an inverse association with electroporation efficiency. This is consistent with earlier reports showing that a further increase in total specific energy did not increase cell permeabilization, indicating that electroporation reaches a plateau once critical electric field thresholds are exceeded (Boussetta et al. 2009; Luengo et al. 2013). Accordingly, although treatments P1–P16 remained within the high‐intensity cluster on Dim‐1, they did not result in further increases in *Z*_p_ despite higher total energy delivery.

**FIGURE 5:**
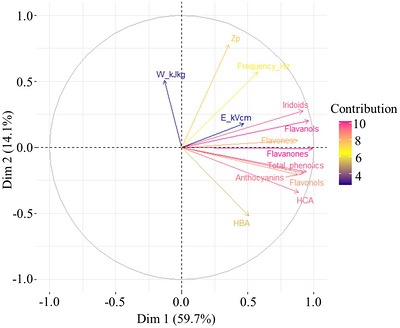
PCA loading plot of variables.

**FIGURE A1:**
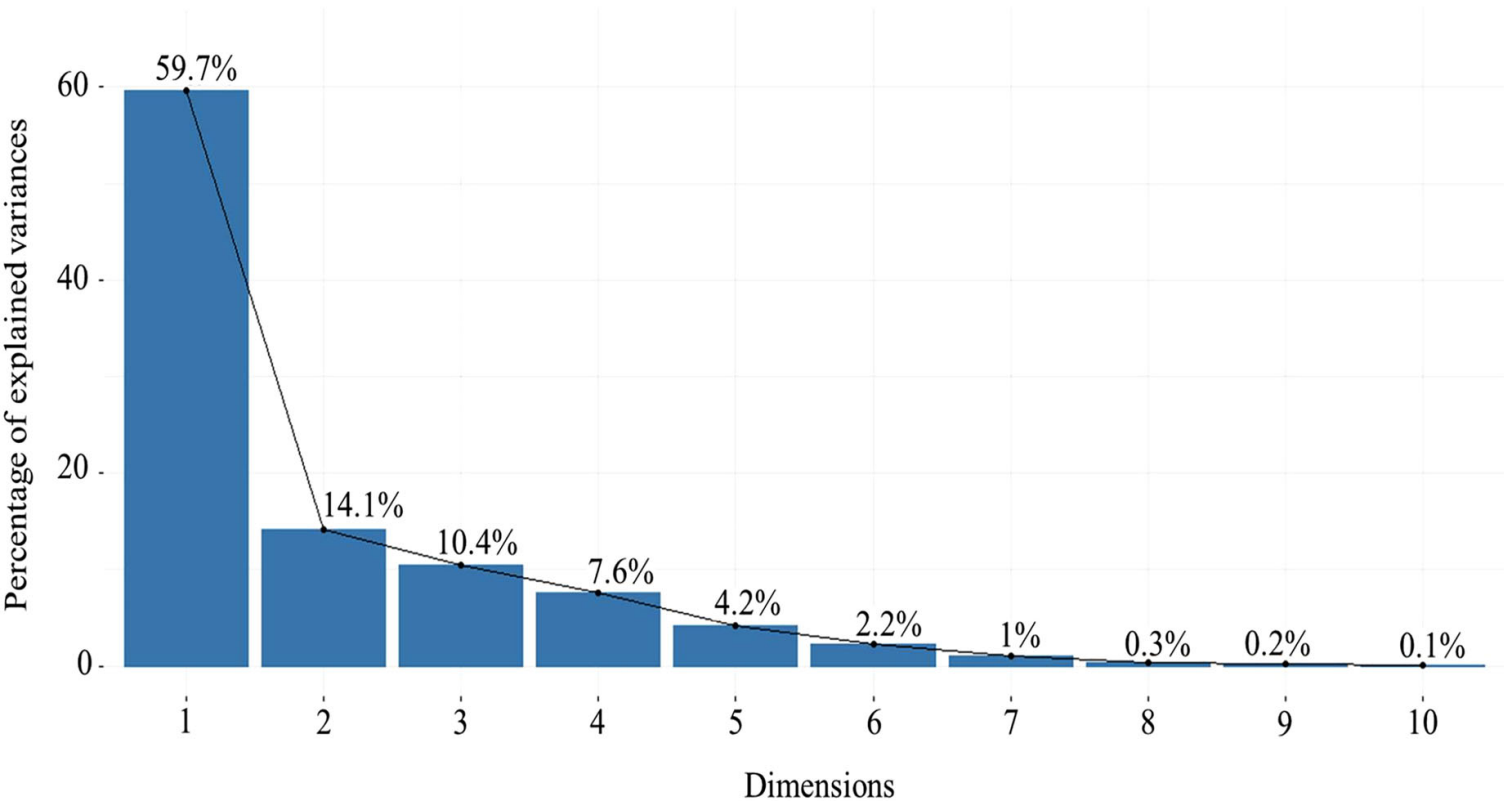
Percentage of variance explained by each principal component.

**FIGURE A2:**
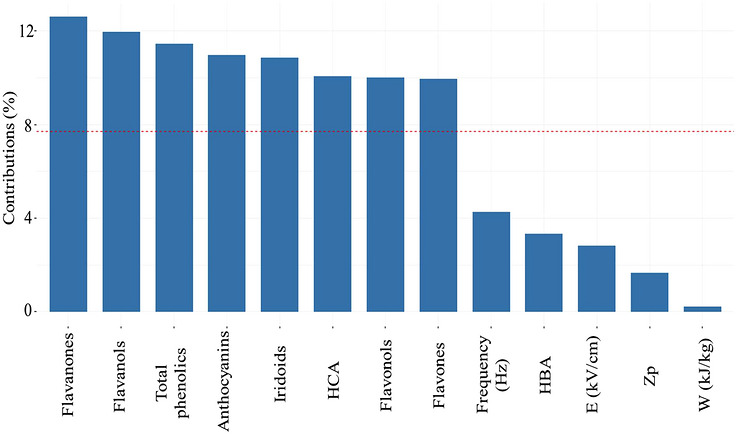
Contribution of variables to dimension 1 (Dim‐1) of PCA. The red dotted line represents the threshold of average contribution. Variables above this line contribute more than average to Dim‐1.

**FIGURE A3:**
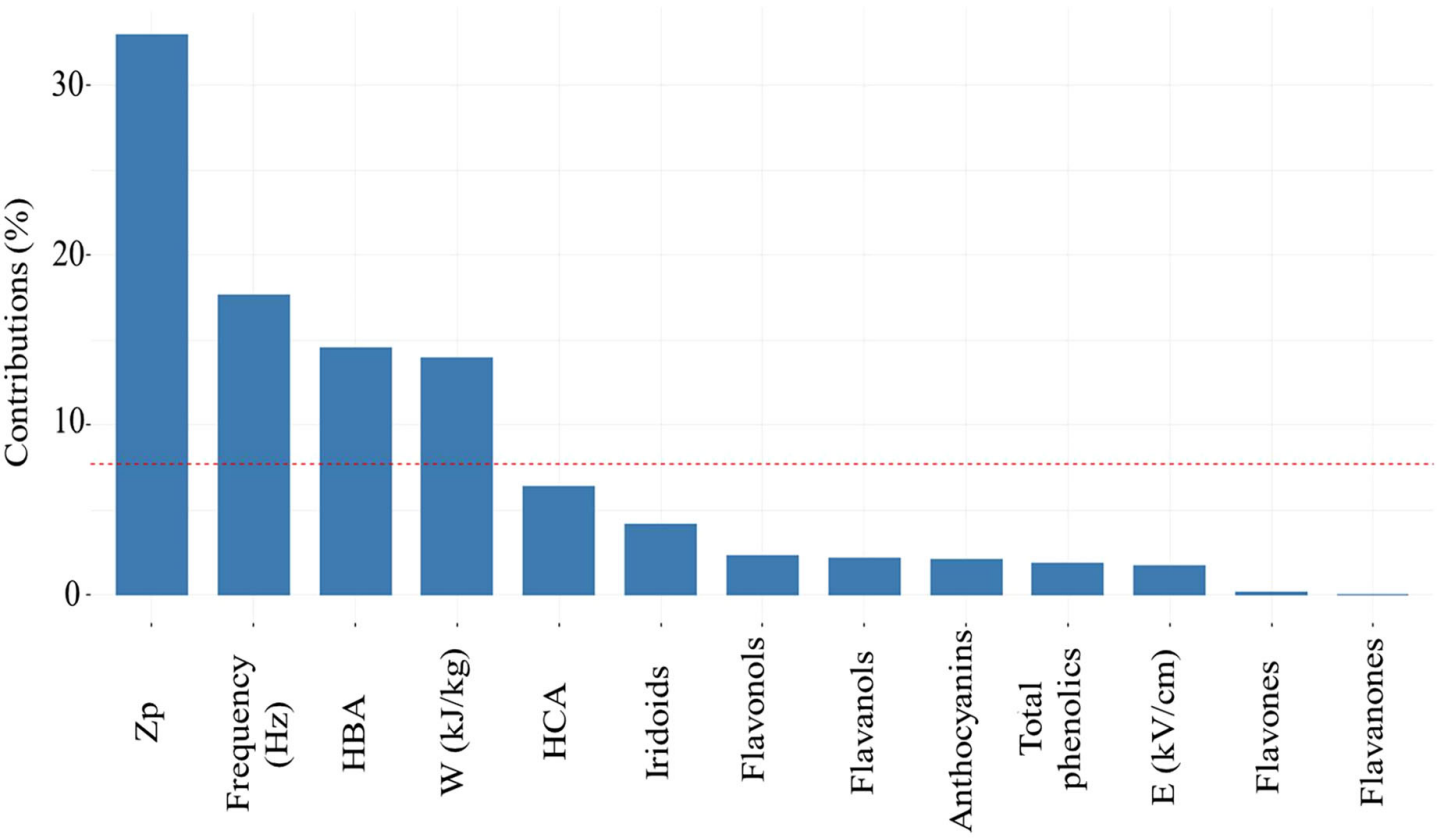
Contribution of variables to dimension 2 (Dim‐2) of PCA. The red dotted line represents the threshold of average contribution. Variables above this line contribute more than average to Dim‐2.

Although most phenolic classes responded coherently to electroporation and clustered strongly along Dim‐1, HBA was positioned closer to Dim‐2 and exhibited a weaker association with the principal electroporation axis, indicating lower sensitivity to membrane permeabilization (Figure 5). This likely reflects structural and localization differences within the pomace matrix, considering that permeabilization of plant tissues is a complex function of the interaction between PEF parameters and material properties (Lamanauskas et al. 2016).

Overall, the PCA indicated that sample discrimination was primarily structured by electric field strength and pulse frequency, which defined the variance accounted for by Dim‐1. Additionally, the close spatial association between *Z*_p_ and the major phenolic groups along Dim‐1 axis supported the cell disintegration as a mechanism that affected the PEF‐assisted phenolic extraction in haskap pomace. Additional PCA grouping analyses according to pulse frequency, specific energy, and electric field strength are provided in Figures S1–S3 in the supplementary material. The scree plot illustrating the percentage of variance explained by each principal component is presented in Figure A1, while the variable contribution plots are presented in Figures A2 and A3, respectively. These analyses were consistent with the PCA loading results by confirming that the electric field strength and pulse frequency were the primary variables contributing to sample discrimination, whereas specific energy showed only a minor contribution to treatment differentiation.

From a mass‐transfer perspective, the observed relationship between *Z*_p_ and phenolic release suggests that extraction enhancement in haskap pomace was affected primarily by changes in membrane transport resistance induced by electroporation. PEF treatment promotes pore formation in cellular membranes, thereby reducing diffusional barriers and facilitating solvent penetration into intracellular regions as well as migration of solubilized compounds toward the extraction medium (Raso et al. 2016; Rrucaj et al. 2024). However, unlike intact tissues commonly investigated in PEF studies, haskap pomace represents a heterogeneous matrix composed of disrupted skins, pulp residues, and seeds generated after juice pressing. Berry pomaces are known to be rich in insoluble dietary fiber fractions, predominantly cellulose, hemicellulose, and lignin, and these structural components may act as physical barriers to mass transfer and limit compound release during extraction (Cacak‐Pietrzak et al. 2025; Jurevičiūtė et al. 2022). Furthermore, previous studies have shown that PEF responses are highly matrix dependent and that structural characteristics of plant materials can influence the extent of cell disintegration achieved under comparable processing conditions (Llavata et al. 2024). Consequently, although membrane permeabilization contributes to enhanced extraction, the overall mass‐transfer process in pomace likely remains partially constrained by matrix resistance. This may explain why increasing total energy input beyond moderate levels did not produce proportional increases in *Z*_p_ or phenolic recovery and supports the concept of electroporation saturation reported previously (Boussetta et al. 2009; Luengo et al. 2013; Shorstkii et al. 2022).

## 4 Conclusions

PEF treatment proved to be an effective method for the phenolic extraction from haskap pomace. Among the tested conditions, treatments applying an electric field strength of 1.5 kV/cm combined with pulse frequencies of 5–10 Hz and moderate specific energy inputs (5–12.3 kJ/kg) consistently produced the highest *Z*_p_ values (0.10–0.12) and the greatest extraction of major phenolic groups, particularly anthocyanins, flavonols, flavanols, and HCAs. Anthocyanins were the most abundant phenolic group in haskap and exhibited the strongest response to PEF, reaching up to a 2.6‐fold increase relative to the control, whereas flavanols and flavonols increased by up to 3.5‐fold and 2.3‐fold, respectively. As a result, concentration of total phenolics ranged from approximately 1293 mg/kg in untreated control samples to 5059 mg/kg in P5‐treated (*E* = 1.5 kV/cm, *f* = 10 Hz, and *W* = 1 kJ/kg) samples, corresponding to an almost 3.9‐fold increase.

Multivariate analyses further supported these findings. PCA revealed that electric field strength and pulse frequency were the primary factors structuring sample discrimination along Dim‐1, which explained 59.7% of the total variance. In contrast, results of the regression analysis for specific energy indicated predominantly nonsignificant or negative coefficients, implying that specific energy was not a statistically meaningful predictor of phenolic extractability under the tested conditions.

From an industrial perspective, the findings indicate that PEF may represent a promising pretreatment strategy for the valorization of haskap processing residues. However, the relatively low *Z*_p_ values observed in this study suggest that further optimization remains necessary before industrial implementation, particularly considering the heterogeneous and fiber‐rich structure of haskap pomace. Future research should, therefore, investigate broader PEF operating conditions, including electric field strength, pulse duration, and waveform characteristics, as well as evaluate continuous‐flow processing configurations, techno‐economic feasibility, compound stability, and bioaccessibility under industrially relevant conditions. Furthermore, integrating structural characterization approaches (e.g., SEM and FTIR) alongside antioxidant activity assessment would provide additional insight into PEF‐induced microstructural changes and their relationship with phenolic recovery and functional properties of haskap pomace extracts.

## Author Contributions

**Alema Puzovic**: conceptualization, investigation, writing – original draft, validation, formal analysis, writing – review and editing, data curation, visualization. **Massimiliano Rinaldi**: writing – review and editing, supervision, resources. **Maja Mikulic‐Petkovsek**: validation, supervision, methodology, writing – review and editing, project administration, funding acquisition.

## Funding

This work received funding from the European Union Horizon 2020 research and innovation program under the Marie Skłodowska‐Curie grant agreement (no. 956257). The authors also acknowledge the financial support of Slovenian Research and Innovation Agency (ARIS) within the research program Horticulture (P4‐0013).

## Conflicts of Interest

The authors declare no conflicts of interest.

## Supporting information




**Supplementary Materials**: jfds71292‐sup‐0001‐SuppMat.docx

## Data Availability

The datasets generated and/or analyzed during the study are available from the corresponding author upon request.

## 1

**TABLE A1:** Phenolic compounds concentration in haskap pomace.

Treatment	Anthocyanins	Iridoids	Flavones	Flavanones	Flavonols	Flavanols	HCA	HBA
C	1437.50 ± 193.33^a^	9.23 ± 1.93^a^	0.59 ± 0.10^a^	0.66 ± 0.13^a^	36.40 ± 7.90^a^	116.36 ± 29.16^a^	128.73 ± 30.52^a^	1.11 ± 0.21^a^
F	1529.46 ± 488.84^a^	12.28 ± 3.28^ab^	1.39 ± 0.35^abc^	0.72 ± 0.22^a^	46.94 ± 13.97^ab^	173.47 ± 36.82^ab^	162.69 ± 40.80^a^	2.04 ± 0.42^ab^
P1	2483.16 ± 451.00^ab^	14.03 ± 4.86^ac^	1.79 ± 0.24^cd^	0.89 ± 0.08^ab^	61.03 ± 8.23^ab^	220.12 ± 22.47^ac^	214.48 ± 28.51^ab^	2.28 ± 0.23^ab^
P2	2152.76 ± 486.18^ab^	13.86 ± 3.59^ac^	1.63 ± 0.33^cd^	0.73 ± 0.10^a^	54.77 ± 11.29^ab^	200.56 ± 36.23^aef^	182.51 ± 35.17^ab^	1.92 ± 0.45^ab^
P3	2260.64 ± 528.48^ab^	14.15 ± 2.87^aci^	1.41 ± 0.16^abc^	0.73 ± 0.01^a^	48.32 ± 6.91^ab^	179.55 ± 25.56^ae^	161.59 ± 28.90^a^	2.12 ± 0.29^ab^
P4	2404.82 ± 311.11^ab^	20.41 ± 3.41^ae^	2.00 ± 0.24^cd^	0.94 ± 0.06^ab^	68.40 ± 8.69^ab^	239.93 ± 23.67^acg^	225.09 ± 28.90^ab^	2.90 ± 0.35^b^
P5	3715.08 ± 287.49^b^	29.54 ± 2.82^dei^	2.04 ± 0.12^cd^	1.29 ± 0.1^bc^	81.47 ± 7.39^b^	343.40 ± 34.20^cd^	294.08 ± 21.61^b^	2.72 ± 0.21^bd^
P6	3310.86 ± 214.55^bc^	24.86 ± 4.34^bcegh^	1.86 ± 0.21^cd^	1.13 ± 0.2^ab^	70.25 ± 10.63^ab^	294.71 ± 39.37^bcde^	236.74 ± 34.79^ab^	1.53 ± 0.17^acd^
P7	2601.71 ± 577.48^ab^	14.97 ± 1.57^acfi^	1.63 ± 0.10^cd^	0.93 ± 0.05^ab^	58.60 ± 3.78^ab^	234.76 ± 21.98^acg^	190.89 ± 21.39^ab^	1.25 ± 0.08^ac^
P8	1949.06 ± 403.41^ac^	17.57 ± 4.02^ae^	1.36 ± 0.15^ac^	0.75 ± 0.07^ac^	51.05 ± 7.54^ab^	226.77 ± 39.36^acg^	173.55 ± 25.37^ab^	1.08 ± 0.18^a^
P9	2266.89 ± 204.59^ab^	22.85 ± 0.49^aeh^	1.58 ± 0.05^cd^	0.81 ± 0.02^ab^	54.31 ± 1.55^ab^	253.04 ± 7.74^acg^	196.93 ± 5.87^ab^	1.32 ± 0.06^ac^
P10	2818.96 ± 115.71^ab^	27.83 ± 0.73^cde^	1.82 ± 0.12^cd^	1.01 ± 0.05^ab^	62.38 ± 3.48^ab^	278.71 ± 5.85^bcde^	197.12 ± 7.15^ab^	1.76 ± 0.20^ab^
P11	2944.35 ± 120.53^ab^	30.10 ± 3.06^def^	1.96 ± 0.15^cd^	1.10 ± 0.11^ab^	71.22 ± 6.52^ab^	316.24 ± 27.72^cde^	229.57 ± 13.03^ab^	2.47 ± 0.17^bc^
P12	2949.50 ± 167.58^ab^	32.41 ± 3.13^de^	2.16 ± 0.13^cd^	1.17 ± 0.08^ab^	79.99 ± 3.52^b^	330.14 ± 23.26^cdf^	233.91 ± 10.07^ab^	2.41 ± 0.13^bc^
P13	3113.37 ± 288.08^ab^	39.21 ± 1.17^dg^	2.30 ± 0.15^cd^	1.29 ± 0.08^bc^	68.16 ± 2.11^ab^	398.41 ± 9.70^d^	249.31 ± 12.35^ab^	2.21 ± 0.21^ab^
P14	3545.60 ± 221.19^bc^	37.67 ± 1.24^dh^	2.02 ± 0.12^cd^	1.18 ± 0.07^ab^	68.66 ± 3.22^ab^	363.33 ± 11.71^dg^	228.43 ± 7.62^ab^	1.73 ± 0.16^ab^
P15	3383.88 ± 282.44^bc^	40.49 ± 3.90^d^	2.36 ± 0.23^bd^	1.34 ± 0.13^b^	70.84 ± 6.16^ab^	401.97 ± 27.42^d^	233.57 ± 21.48^ab^	2.19 ± 0.15^ab^
P16	2820.86 ± 123.12^ab^	31.64 ± 2.71^de^	2.52 ± 0.12^d^	1.15 ± 0.05^ab^	66.75 ± 3.57^ab^	349.90 ± 19.02^cd^	227.58 ± 12.79^ab^	2.11 ± 0.16^ab^
*p*	0.000	0.000	0.000	0.000	0.003	0.000	0.003	0.000

*Note*: The values are means of four repetitions. Mean values followed by different letters in the column are significantly different according to Tukey's test (*p *≤ 0.05).

**TABLE A2:** Regression coefficients for pulsed electric field (PEF) predictors.

Compound	Term	Estimate	±SE	*p*	*R* ^2^
Anthocyanins	Intercept	1148.85	543.57	0.039	0.201
Anthocyanins	*E* (kV/cm)	1039.84	354.63	0.005	0.201
Anthocyanins	*f* (Hz)	74.13	35.46	0.041	0.201
Anthocyanins	*W* (kJ/kg)	−29.31	19.96	0.147	0.201
Iridoids	Intercept	−13.5	5.26	0.013	0.592
Iridoids	*E* (kV/cm)	15.08	3.43	0.000	0.592
Iridoids	*f* (Hz)	2.82	0.34	0.000	0.592
Iridoids	*W* (kJ/kg)	−0.11	0.19	0.577	0.592
Flavones	Intercept	0.76	0.31	0.016	0.238
Flavones	*E* (kV/cm)	0.44	0.2	0.035	0.238
Flavones	*f* (Hz)	0.08	0.02	0.000	0.238
Flavones	*W* (kJ/kg)	0.0	0.01	0.706	0.238
Flavanones	Intercept	0.22	0.17	0.185	0.334
Flavanones	*E* (kV/cm)	0.42	0.11	0.000	0.334
Flavanones	*f* (Hz)	0.04	0.01	0.000	0.334
Flavanones	*W* (kJ/kg)	0.0	0.01	0.456	0.334
Flavonols	Intercept	45.21	11.26	0.000	0.065
Flavonols	*E* (kV/cm)	8.84	7.35	0.234	0.065
Flavonols	*f* (Hz)	1.21	0.73	0.105	0.065
Flavonols	*W* (kJ/kg)	−0.08	0.41	0.849	0.065
Flavanols	Intercept	−27.38	43.38	0.530	0.547
Flavanols	*E* (kV/cm)	148.74	28.3	0.000	0.547
Flavanols	*f* (Hz)	18.8	2.83	0.000	0.547
Flavanols	*W* (kJ/kg)	−1.41	1.59	0.381	0.547
HCA	Intercept	150.51	36.71	0.000	0.120
HCA	*E* (kV/cm)	48.24	23.95	0.048	0.120
HCA	*f* (Hz)	2.94	2.39	0.225	0.120
HCA	*W* (kJ/kg)	−2.18	1.35	0.110	0.120
HBA	Intercept	2.62	0.49	0.000	0.057
HBA	*E* (kV/cm)	−0.59	0.32	0.070	0.057
HBA	*f* (Hz)	0.01	0.03	0.758	0.057
HBA	*W* (kJ/kg)	0.01	0.02	0.758	0.057
Total phenolics	Intercept	1307.3	572.86	0.026	0.259
Total phenolics	*E* (kV/cm)	1260.99	373.73	0.001	0.259
Total phenolics	*f* (Hz)	100.03	37.37	0.010	0.259
Total phenolics	*W* (kJ/kg)	−33.08	21.04	0.121	0.259
