# Supplementary material for: Pulsed Electric Field‐Assisted Recovery of Phenolic Compounds From Haskap (Lonicera caerulea L.) Pomace: Influence of Processing Parameters and Cell Disintegration
Source: J Food Sci. 2026 Jul 13;91(7):e71292. doi: 10.1111/1750-3841.71292 (PMC13359348; doi:10.1111/1750-3841.71292)
Supplement: Supplementary file 1 — Supplementary Materials: jfds71292‐sup‐0001‐SuppMat.docx [file JFDS-91-0-s001.docx]

**Table S 1:** Complete experimental matrix and replication structure of PEF treatments applied to haskap pomace.

| Sample name and replicate | Electric field strength (kV/cm) | Pulse width (±µs) | Relax time | Frequency (Hz) | No of pulses | Specific energy (kJ/kg) | Temperature before the treatment (°C) | Temperature after the treatment (°C) | Conductivity before the treatment (mS) | Conductivity after the treatment (mS) |
| --- | --- | --- | --- | --- | --- | --- | --- | --- | --- | --- |
| P 1.1 | 1 | 10 | 10 | 5 | 140 | 1.0 | 31 | 34 | 4.4 | 4.48 |
| P 1.2 | 1 | 10 | 10 | 5 | 140 | 1.0 | 33 | 34 | 4.41 | 4.45 |
| P 1.3 | 1 | 10 | 10 | 5 | 140 | 1.0 | 34 | 34 | 4.48 | 4.5 |
| P 1.4 | 1 | 10 | 10 | 5 | 140 | 1.0 | 33 | 34 | 4.53 | 4.7 |
| P 2.1 | 1 | 10 | 10 | 5 | 720 | 5.0 | 32 | 34 | 4.44 | 4.49 |
| P 2.2 | 1 | 10 | 10 | 5 | 720 | 5.0 | 32 | 32 | 4.45 | 4.57 |
| P 2.3 | 1 | 10 | 10 | 5 | 720 | 5.0 | 34 | 34 | 4.48 | 4.59 |
| P 2.4 | 1 | 10 | 10 | 5 | 720 | 5.0 | 32 | 34 | 4.45 | 4.54 |
| P 3.1 | 1 | 10 | 10 | 5 | 1430 | 10.3 | 32 | 36 | 4.47 | 5.13 |
| P 3.2 | 1 | 10 | 10 | 5 | 1430 | 10.3 | 33 | 40 | 4.57 | 5.52 |
| P 3.3 | 1 | 10 | 10 | 5 | 1430 | 10.3 | 33 | 39 | 4.5 | 5.5 |
| P 3.4 | 1 | 10 | 10 | 5 | 1430 | 10.3 | 33 | 40 | 4.52 | 5.05 |
| P 4.1 | 1 | 10 | 10 | 5 | 1750 | 12.3 | 32 | 38 | 4.47 | 6.42 |
| P 4.2 | 1 | 10 | 10 | 5 | 1750 | 12.3 | 33 | 40 | 4.59 | 6.19 |
| P 4.3 | 1 | 10 | 10 | 5 | 1750 | 12.3 | 33 | 39 | 4.65 | 6.71 |
| P 4.4 | 1 | 10 | 10 | 5 | 1750 | 12.3 | 34 | 40.5 | 4.61 | 6.36 |
| P 5.1 | 1.5 | 10 | 10 | 5 | 30 | 1.0 | 30 | 33 | 4.57 | 5.67 |
| P 5.2 | 1.5 | 10 | 10 | 5 | 30 | 1.0 | 29 | 32 | 4.52 | 4.68 |
| P 5.3 | 1.5 | 10 | 10 | 5 | 30 | 1.0 | 29 | 31 | 4.58 | 5.46 |
| P 5.4 | 1.5 | 10 | 10 | 5 | 30 | 1.0 | 29 | 34 | 4.55 | 5.54 |
| P 6.1 | 1.5 | 10 | 10 | 5 | 150 | 5.0 | 29 | 41 | 4.55 | 4.66 |
| P 6.2 | 1.5 | 10 | 10 | 5 | 150 | 5.0 | 29 | 40 | 4.42 | 4.43 |
| P 6.3 | 1.5 | 10 | 10 | 5 | 150 | 5.0 | 28 | 40.5 | 4.44 | 4.63 |
| P 6.4 | 1.5 | 10 | 10 | 5 | 150 | 5.0 | 28 | 42 | 4.4 | 4.46 |
| P 7.1 | 1.5 | 10 | 10 | 5 | 300 | 10.3 | 28 | 34 | 4.38 | 4.85 |
| P 7.2 | 1.5 | 10 | 10 | 5 | 300 | 10.3 | 28 | 34 | 4.35 | 4.72 |
| P 7.3 | 1.5 | 10 | 10 | 5 | 300 | 10.3 | 28 | 34 | 4.41 | 4.79 |
| P 7.4 | 1.5 | 10 | 10 | 5 | 300 | 10.3 | 28 | 34 | 4.74 | 5.17 |
| P 8.1 | 1.5 | 10 | 10 | 5 | 370 | 12.3 | 30 | 38 | 4.33 | 5.37 |
| P 8.2 | 1.5 | 10 | 10 | 5 | 370 | 12.3 | 31 | 40 | 4.29 | 5.29 |
| P 8.3 | 1.5 | 10 | 10 | 5 | 370 | 12.3 | 28 | 34 | 4.32 | 5.04 |
| P 8.4 | 1.5 | 10 | 10 | 5 | 370 | 12.3 | 31 | 38.5 | 4.44 | 5.52 |
| P 9.91 | 1 | 10 | 10 | 10 | 70 | 1.0 | 32 | 37 | 4.32 | 4.84 |
| P 9.2 | 1 | 10 | 10 | 10 | 70 | 1.0 | 29 | 33 | 4.3 | 5.19 |
| P 9.3 | 1 | 10 | 10 | 10 | 70 | 1.0 | 29 | 34 | 4.29 | 5.53 |
| P 9.4 | 1 | 10 | 10 | 10 | 70 | 1.0 | 28 | 33 | 4.37 | 5.38 |
| P 10.1 | 1 | 10 | 10 | 10 | 380 | 5.0 | 27 | 27 | 4.41 | 4.44 |
| P 10.2 | 1 | 10 | 10 | 10 | 380 | 5.0 | 27 | 28 | 4.44 | 4.55 |
| P 10.3 | 1 | 10 | 10 | 10 | 380 | 5.0 | 27 | 28 | 4.3 | 4.45 |
| P 10.4 | 1 | 10 | 10 | 10 | 380 | 5.0 | 27 | 28 | 4.33 | 4.29 |
| P 11.1 | 1 | 10 | 10 | 10 | 730 | 10.3 | 27 | 33 | 4.34 | 4.84 |
| P 11.2 | 1 | 10 | 10 | 10 | 730 | 10.3 | 28 | 33 | 4.31 | 4.68 |
| P 11.3 | 1 | 10 | 10 | 10 | 730 | 10.3 | 28 | 32 | 4.32 | 4.68 |
| P 11.4 | 1 | 10 | 10 | 10 | 730 | 10.3 | 28 | 33 | 4.34 | 4.67 |
| P 12.1 | 1 | 10 | 10 | 10 | 890 | 12.3 | 29 | 36 | 4.46 | 5.35 |
| P 12.2 | 1 | 10 | 10 | 10 | 890 | 12.3 | 28 | 36.5 | 4.36 | 5.13 |
| P 12.3 | 1 | 10 | 10 | 10 | 890 | 12.3 | 28 | 37 | 4.37 | 5.06 |
| P 12.4 | 1 | 10 | 10 | 10 | 890 | 12.3 | 29 | 38 | 4.34 | 5.27 |
| P 13.1 | 1.5 | 10 | 10 | 10 | 15 | 1.0 | 23 | 25.6 | 4.37 | 5.46 |
| P 13.2 | 1.5 | 10 | 10 | 10 | 15 | 1.0 | 23.8 | 25.3 | 4.34 | 5.29 |
| P 13.3 | 1.5 | 10 | 10 | 10 | 15 | 1.0 | 24.4 | 25.5 | 4.33 | 5.36 |
| P 13.4 | 1.5 | 10 | 10 | 10 | 15 | 1.0 | 24.5 | 25.4 | 4.35 | 5.17 |
| P 14.1 | 1.5 | 10 | 10 | 10 | 75 | 5.0 | 24.7 | 29.5 | 4.34 | 4.44 |
| P 14.2 | 1.5 | 10 | 10 | 10 | 75 | 5.0 | 25.2 | 30.3 | 4.34 | 4.45 |
| P 14.3 | 1.5 | 10 | 10 | 10 | 75 | 5.0 | 26 | 34 | 4.33 | 4.44 |
| P 14.4 | 1.5 | 10 | 10 | 10 | 75 | 5.0 | 25.6 | 35 | 4.37 | 4.42 |
| P 15.1 | 1.5 | 10 | 10 | 10 | 150 | 10.3 | 29 | 37 | 4.32 | 4.46 |
| P 15.2 | 1.5 | 10 | 10 | 10 | 150 | 10.3 | 28 | 36 | 4.34 | 4.77 |
| P 15.3 | 1.5 | 10 | 10 | 10 | 150 | 10.3 | 28 | 38 | 4.33 | 4.49 |
| P 15.4 | 1.5 | 10 | 10 | 10 | 150 | 10.3 | 29 | 38 | 4.34 | 4.48 |
| P 16.1 | 1.5 | 10 | 10 | 10 | 185 | 12.3 | 25 | 34 | 4.33 | 5.21 |
| P 16.2 | 1.5 | 10 | 10 | 10 | 185 | 12.3 | 27 | 37 | 4.32 | 5.26 |
| P 16.3 | 1.5 | 10 | 10 | 10 | 185 | 12.3 | 26 | 36 | 4.35 | 5.33 |
| P 16.4 | 1.5 | 10 | 10 | 10 | 185 | 12.3 | 28 | 35 | 4.33 | 5.34 |

**Table S 2:**Concentration of individual anthocyanins in haskap pomace samples (mg/kg). Values represent means of four independent repetitions (n = 4). C = control sample; F = frozen sample; P1-P16 = samples treated using pulsed electric field (PEF) under different processing conditions. Mean values followed by different superscript letters within each compound are significantly different according to Tukey’s HSD test (*p* ≤ 0.05).

| **Compound** | **C** | **F** | **P1** | **P2** | **P3** | **P4** | **P5** | **P6** | **P7** | **P8** | **P9** | **P10** | **P11** | **P12** | **P13** | **P14** | **P15** | **P16** |
| --- | --- | --- | --- | --- | --- | --- | --- | --- | --- | --- | --- | --- | --- | --- | --- | --- | --- | --- |
| **Cyanidin-3,5-diglucoside** | 29.83 ± 7.60ᵃ | 40.69 ± 9.73ᵃᵇ | 41.84 ± 6.50ᵃᵇ | 37.70 ± 6.95ᵃᶜ | 27.94 ± 6.92ᵃ | 41.07 ± 7.66ᵃᵇ | 71.58 ± 9.16ᵇᶜ | 51.38 ± 6.07ᵃᵇ | 53.36 ± 9.39ᵃᵇ | 37.86 ± 8.29ᵃᶜ | 48.35 ± 4.80ᵃᵇ | 43.74 ± 2.67ᵃᵇ | 51.88 ± 4.53ᵃᵇ | 57.02 ± 4.02ᵃᵇ | 65.73 ± 3.95ᵇᶜ | 69.01 ± 3.46ᵇᶜ | 72.81 ± 7.93ᵇ | 59.04 ± 4.66ᵃᵇ |
| **Cyanidin-3-O-glucoside** | 1127.75 ± 181.12ᵃ | 1735.03 ± 454.35ᵃᵇᶜ | 2580.46 ± 345.89ᶜᵈ | 1873.77 ± 423.06ᵃᵈ | 1766.30 ± 336.27ᵃᵇᶜ | 2595.40 ± 335.04ᶜᵈ | 3303.80 ± 244.16ᵈ | 2713.02 ± 453.41ᶜᵈ | 1560.28 ± 171.72ᵃᶜ | 1812.51 ± 322.68ᵃᵇᶜ | 2308.82 ± 58.28ᵃᵈ | 2477.51 ± 98.27ᵃᵈ | 2550.60 ± 150.47ᵃᵈ | 2512.48 ± 120.26ᵃᵈ | 3145.97 ± 220.24ᵇᵈ | 2724.66 ± 146.41ᶜᵈ | 2601.44 ± 343.32ᶜᵈ | 2557.69 ± 124.78ᵃᵈ |
| **Cyanidin-3-O-rutinoside** | 23.98 ± 4.52ᵃ | 29.44 ± 7.08ᵃᵇ | 47.94 ± 5.36ᵃᶜ | 41.72 ± 9.97ᵃᵈ | 36.66 ± 6.13ᵃᵈ | 55.07 ± 8.79ᵃᶜ | 80.91 ± 4.09ᶜ | 56.20 ± 6.01ᵃᶜ | 55.12 ± 9.81ᵃᶜ | 51.86 ± 4.85ᵃᶜ | 63.02 ± 4.37ᵇᶜᵈ | 67.54 ± 2.63ᶜᵈ | 66.66 ± 6.95ᶜᵈ | 67.95 ± 2.23ᶜᵈ | 77.89 ± 7.05ᶜ | 81.56 ± 4.83ᶜ | 66.72 ± 10.95ᶜᵈ | 58.37 ± 8.08ᵃᶜ |
| **Cyanidin-3-O-rhamnosyl-hexoside** | 2.92 ± 0.46ᵃ | 2.29 ± 0.26ᵃ | 5.09 ± 1.68ᵃ | 3.94 ± 1.19ᵃ | 3.78 ± 0.63ᵃ | 3.80 ± 0.30ᵃ | 4.05 ± 0.10ᵃ | 3.32 ± 0.34ᵃ | 2.64 ± 0.24ᵃ | 2.46 ± 0.47ᵃ | 2.83 ± 0.08ᵃ | 4.07 ± 0.62ᵃ | 3.86 ± 0.32ᵃ | 4.09 ± 0.21ᵃ | 4.95 ± 0.97ᵃ | 5.74 ± 0.72ᵃ | 5.19 ± 0.70ᵃ | 4.67 ± 0.28ᵃ |
| **Delphinidin-3-O-rutinoside** | 0.44 ± 0.05ᵃ | 0.45 ± 0.15ᵃ | 1.35 ± 0.31ᵃᵇ | 1.60 ± 0.16ᵃᶜ | 2.05 ± 0.43ᵃᵈᶠ | 2.69 ± 0.51ᵇᶜᵈᵉ | 3.23 ± 0.22ᵈᵉ | 3.02 ± 0.48ᶜᵈᵉ | 1.72 ± 0.17ᵃᵈ | 1.93 ± 0.45ᵃᵈᶠ | 2.45 ± 0.09ᵇᶜᵈᵉ | 2.92 ± 0.13ᵇᶜᵈᵉ | 3.23 ± 0.34ᵈᵉ | 3.46 ± 0.31ᵉ | 3.83 ± 0.26ᵉ | 3.98 ± 0.16ᵉ | 3.95 ± 0.43ᵉ | 3.24 ± 0.35ᵈᵉ |
| **Pelargonidin-3-O-glucoside** | 54.10 ± 10.32ᵃ | 57.76 ± 11.69ᵃᵇ | 121.07 ± 27.51ᵃᶜᵈᵉ | 78.50 ± 13.26ᵃᶜ | 83.57 ± 14.99ᵃᶜᵍ | 135.18 ± 18.14ᵇᶜᵈᵉᶠ | 212.70 ± 17.78ᶠ | 153.63 ± 16.33ᶜᵈᶠ | 114.57 ± 17.47ᵃᵈᵍ | 124.05 ± 23.60ᵃᶜᵈᵉ | 144.71 ± 4.69ᶜᵈᶠ | 135.32 ± 3.30ᵇᶜᵈᵉᶠ | 158.36 ± 20.31ᵉᶠᵍ | 169.06 ± 9.99ᵈᶠ | 198.11 ± 13.06ᵉᶠ | 196.83 ± 8.72ᵉᶠ | 203.34 ± 10.72ᶠ | 169.06 ± 11.14ᵈᶠ |
| **Pelargonidin-dihexoside** | 0.31 ± 0.15ᵃᵇ | 0.13 ± 0.02ᵃ | 0.38 ± 0.10ᵃᵇ | 0.35 ± 0.08ᵃᵇ | 0.61 ± 0.15ᵇ | 0.58 ± 0.17ᵇᶜ | 0.34 ± 0.01ᵃᵇ | 0.32 ± 0.02ᵃᵇ | 0.23 ± 0.02ᵃᵇ | 0.35 ± 0.04ᵃᵇ | 0.25 ± 0.02ᵃᵇ | 0.22 ± 0.01ᵃᶜ | 0.25 ± 0.04ᵃᵇ | 0.23 ± 0.01ᵃᵇ | 0.24 ± 0.03ᵃᵇ | 0.24 ± 0.01ᵃᵇ | 0.35 ± 0.05ᵃᵇ | 0.41 ± 0.05ᵃᵇ |
| **Peonidin-3,5-dihexoside** | 15.49 ± 7.35ᵃᵇ | 6.23 ± 1.11ᵃ | 18.61 ± 5.08ᵃᵇ | 17.57 ± 3.83ᵃᵇ | 30.35 ± 7.43ᵇ | 28.77 ± 8.32ᵇᶜ | 17.02 ± 0.68ᵃᵇ | 15.89 ± 0.97ᵃᵇ | 11.33 ± 1.08ᵃᵇ | 17.58 ± 1.94ᵃᵇ | 12.40 ± 0.86ᵃᵇ | 10.75 ± 0.61ᵃᶜ | 12.39 ± 1.94ᵃᵇ | 11.40 ± 0.71ᵃᵇ | 11.84 ± 1.33ᵃᵇ | 11.70 ± 0.36ᵃᵇ | 17.34 ± 2.48ᵃᵇ | 20.47 ± 2.70ᵃᵇ |
| **Peonidin-3-O-glucoside** | 36.90 ± 5.68ᵃ | 56.29 ± 14.11ᵃᵇ | 78.71 ± 10.60ᵃᶜ | 56.86 ± 14.69ᵃᵇ | 50.82 ± 16.21ᵃᵈ | 86.46 ± 11.81ᵃᶜ | 109.05 ± 9.68ᵇᶜ | 87.73 ± 13.06ᵃᶜ | 57.38 ± 6.70ᵃᵇ | 62.58 ± 12.18ᵃᵇ | 77.18 ± 1.88ᵃᶜ | 91.12 ± 3.07ᵇᶜᵈ | 96.36 ± 8.91ᵇᶜᵈ | 101.83 ± 9.16ᵇᶜᵈ | 122.39 ± 3.99ᶜ | 119.39 ± 3.55ᶜ | 125.64 ± 15.18ᶜ | 98.62 ± 7.79ᵇᶜᵈ |
| **Peonidin-3-O-rutinoside** | 1.53 ± 0.18ᵃ | 1.57 ± 0.52ᵃ | 4.69 ± 1.08ᵃᵇ | 5.57 ± 0.57ᵃᶜ | 7.11 ± 1.49ᵃᵈᶠ | 9.35 ± 1.77ᵇᶜᵈᵉ | 11.23 ± 0.77ᵈᵉ | 10.49 ± 1.68ᶜᵈᵉ | 5.98 ± 0.58ᵃᵈ | 6.69 ± 1.57ᵃᵈᶠ | 8.52 ± 0.31ᵇᶜᵈᵉ | 10.13 ± 0.45ᵇᶜᵈᵉ | 11.23 ± 1.16ᵈᵉ | 12.03 ± 1.08ᵉᶠ | 13.31 ± 0.89ᵉ | 13.84 ± 0.56ᵉ | 13.73 ± 1.49ᵉ | 11.27 ± 1.22ᵈᵉ |

**Table S 3:** Concentration of individual iridoids in haskap pomace samples (mg/kg). Values represent means of four independent repetitions (n = 4). C = control sample; F = frozen sample; P1-P16 = samples treated using pulsed electric field (PEF) under different processing conditions. Mean values followed by different superscript letters within each compound are significantly different according to Tukey’s HSD test (*p* ≤ 0.05).

| **Compound** | **C** | **F** | **P1** | **P2** | **P3** | **P4** | **P5** | **P6** | **P7** | **P8** | **P9** | **P10** | **P11** | **P12** | **P13** | **P14** | **P15** | **P16** |
| --- | --- | --- | --- | --- | --- | --- | --- | --- | --- | --- | --- | --- | --- | --- | --- | --- | --- | --- |
| **Loganin-7-O-pentoside** | 12.86 ± 2.80ᵃ | 17.11 ± 4.69ᵃᵇ | 19.27 ± 6.63ᵃᶜ | 19.23 ± 5.02ᵃᶜ | 19.62 ± 4.03ᵃᶜ | 28.23 ± 4.71ᵃᶜᶠ | 40.83 ± 4.18ᶜᵈ | 34.46 ± 6.12ᵃᶜᵈ | 20.72 ± 2.03ᵃᶜᵉ | 24.52 ± 5.78ᵃᶜᶠ | 31.71 ± 0.88ᵃᶜᶠᵍ | 38.55 ± 0.89ᵇᶜᵈ | 41.68 ± 4.26ᵈᵉᶠ | 44.32 ± 4.24ᵈᶠ | 54.20 ± 2.03ᵈ | 52.11 ± 2.16ᵈᵍ | 55.99 ± 5.62ᵈ | 43.56 ± 3.24ᵈᶠ |

**Table S 4:** Concentration of individual flavones in haskap pomace samples (mg/kg). Values represent means of four independent repetitions (n = 4). C = control sample; F = frozen sample; P1-P16 = samples treated using pulsed electric field (PEF) under different processing conditions. Mean values followed by different superscript letters within each compound are significantly different according to Tukey’s HSD test (*p* ≤ 0.05).

| **Compound** | **C** | **F** | **P1** | **P2** | **P3** | **P4** | **P5** | **P6** | **P7** | **P8** | **P9** | **P10** | **P11** | **P12** | **P13** | **P14** | **P15** | **P16** |
| --- | --- | --- | --- | --- | --- | --- | --- | --- | --- | --- | --- | --- | --- | --- | --- | --- | --- | --- |
| **Diosmetin-O-rhamnosyl-hexoside** | 0.162 ± 0.051ᵃ | 0.163 ± 0.051ᵃ | 0.230 ± 0.029ᵃᵇ | 0.198 ± 0.051ᵃ | 0.211 ± 0.023ᵃᶠ | 0.233 ± 0.020ᵃᵇ | 0.321 ± 0.029ᵃᵇᵉ | 0.256 ± 0.043ᵃᵇᶜ | 0.382 ± 0.020ᵇᵉᶠ | 0.317 ± 0.026ᵃᵇᵉ | 0.238 ± 0.023ᵃᵇ | 0.261 ± 0.012ᵃᵇᶜ | 0.299 ± 0.020ᵃᵇᶜᵈ | 0.391 ± 0.049ᵇᵉ | 0.451 ± 0.018ᵈᵉ | 0.487 ± 0.038ᵉ | 0.428 ± 0.033ᶜᵉ | 0.479 ± 0.037ᵉ |
| **Genistein-O-hydroxyhexoside** | 0.263 ± 0.060ᵃ | 0.942 ± 0.238ᵃᵇ | 1.213 ± 0.171ᵇ | 1.144 ± 0.221ᵇ | 0.947 ± 0.133ᵃᵇ | 1.413 ± 0.182ᵇ | 1.320 ± 0.104ᵇ | 1.211 ± 0.163ᵇ | 0.909 ± 0.063ᵃᵇ | 0.733 ± 0.132ᵃᵇ | 0.841 ± 0.058ᵃᵇ | 0.837 ± 0.085ᵃᵇ | 1.017 ± 0.103ᵇ | 1.120 ± 0.051ᵇ | 0.928 ± 0.068ᵃᵇ | 0.902 ± 0.027ᵃᵇ | 1.184 ± 0.176ᵇ | 1.129 ± 0.142ᵇ |
| **Luteolin-3-O-rutinoside** | 0.168 ± 0.035ᵃ | 0.286 ± 0.064ᵃ | 0.347 ± 0.047ᵃᵇ | 0.283 ± 0.062ᵃ | 0.251 ± 0.036ᵃ | 0.354 ± 0.045ᵃᵇ | 0.393 ± 0.022ᵃᵈ | 0.388 ± 0.067ᵃᵈ | 0.339 ± 0.037ᵃᵇ | 0.306 ± 0.022ᵃᵇ | 0.502 ± 0.074ᵃᵈᵉ | 0.717 ± 0.037ᶜᵈ | 0.637 ± 0.060ᵇᶜᵈ | 0.644 ± 0.078ᵇᶜᵈ | 0.922 ± 0.069ᶜ | 0.634 ± 0.098ᵇᶜᵈ | 0.744 ± 0.150ᶜᵉ | 0.906 ± 0.055ᶜ |
| **Luteolin-O-hexoside-1 (×10^-3^)** | 0.982 ± 0.147ᵃ | 1.409 ± 0.341ᵃᵇ | 1.800 ± 0.245ᵃᵇᶜ | 1.293 ± 0.302ᵃᵇ | 1.187 ± 0.193ᵃᶠ | 1.752 ± 0.226ᵃᵇᶜ | 1.983 ± 0.236ᵃᵇᶜ | 1.792 ± 0.303ᵃᵇᶜ | 1.428 ± 0.066ᵃᵇᵉ | 1.402 ± 0.205ᵃᵇ | 1.615 ± 0.070ᵃᵇᵈ | 2.844 ± 0.230ᶜᵈ | 2.786 ± 0.384ᶜᵈᵉ | 3.086 ± 0.274ᶜ | 2.572 ± 0.254ᵇᶜ | 1.984 ± 0.377ᵃᵇᶜ | 2.437 ± 0.398ᵇᶜᶠ | 2.522 ± 0.161ᵇᶜᶠ |
| **Luteolin-O-hexoside-2 (×10^-5^)** | 2.38 ± 0.49ᵃ | 3.31 ± 0.74ᵃᵇ | 3.71 ± 0.63ᵃᵇ | 5.50 ± 1.24ᵃᵈ | 4.88 ± 0.40ᵃᵈ | 5.72 ± 0.59ᵃᵈ | 7.01 ± 0.13ᵇᵈᵉ | 6.13 ± 0.59ᵃᵈᵉ | 8.17 ± 0.48ᵈᵉᶠ | 6.31 ± 0.38ᵃᵈᵉ | 7.41 ± 0.09ᵇᵈᵉ | 8.92 ± 0.23ᶜᵈ | 9.90 ± 0.53ᶜᵉ | 10.30 ± 0.24ᶜᵉ | 11.63 ± 1.10ᶜᶠ | 12.44 ± 2.17ᶜ | 12.22 ± 0.94ᶜᶠ | 8.33 ± 0.68ᶜᵈ |

**Table S 5:** Concentration of individual flavanones in haskap pomace samples (mg/kg). Values represent means of four independent repetitions (n = 4). C = control sample; F = frozen sample; P1-P16 = samples treated using pulsed electric field (PEF) under different processing conditions. Mean values followed by different superscript letters within each compound are significantly different according to Tukey’s HSD test (*p* ≤ 0.05).

| **Compound** | **C** | **F** | **P1** | **P2** | **P3** | **P4** | **P5** | **P6** | **P7** | **P8** | **P9** | **P10** | **P11** | **P12** | **P13** | **P14** | **P15** | **P16** |
| --- | --- | --- | --- | --- | --- | --- | --- | --- | --- | --- | --- | --- | --- | --- | --- | --- | --- | --- |
| **Naringenin-O-hexoside-1** | 0.0002 ± 0.0000ᵃ | 0.0003 ± 0.0001ᵃᵇ | 0.0004 ± 0.0001ᵃᶜᵈ | 0.0003 ± 0.0001ᵃᵈ | 0.0004 ± 0.0001ᵃᶜᵈ | 0.0005 ± 0.0001ᵇᵈᵉ | 0.0005 ± 0.0000ᵇᵈᵉ | 0.0005 ± 0.0001ᵇᵈᵉ | 0.0004 ± 0.0000ᵃᶜᵈ | 0.0004 ± 0.0000ᵃᶜᵈ | 0.0004 ± 0.0000ᵃᵉ | 0.0006 ± 0.0000ᵈᵉ | 0.0007 ± 0.0001ᶜᵉ | 0.0007 ± 0.0000ᵉ | 0.0006 ± 0.0000ᵈᵉ | 0.0006 ± 0.0000ᵈᵉ | 0.0005 ± 0.0000ᵇᵈᵉ | 0.0005 ± 0.0000ᵇᵈᵉ |
| **Naringenin-O-hexoside-2** | 0.006 ± 0.002ᵃ | 0.007 ± 0.002ᵃ | 0.011 ± 0.001ᵃᵇᶜ | 0.010 ± 0.002ᵃᶜ | 0.012 ± 0.002ᵃᵇᶜ | 0.006 ± 0.001ᵃ | 0.014 ± 0.002ᵃᵈ | 0.008 ± 0.002ᵃ | 0.013 ± 0.001ᵃᵇᶜ | 0.011 ± 0.001ᵃᵇᶜ | 0.009 ± 0.001ᵃᶜ | 0.012 ± 0.001ᵃᵇᶜ | 0.018 ± 0.002ᶜᵈ | 0.030 ± 0.004ᵉ | 0.024 ± 0.003ᵈᵉ | 0.020 ± 0.003ᵇᵈ | 0.023 ± 0.002ᵈᵉ | 0.023 ± 0.001ᵈᵉ |
| **Naringenin-O-hexoside-3** | 0.526 ± 0.119ᵃᵇ | 0.539 ± 0.213ᵃᵇ | 0.580 ± 0.071ᵃᵇ | 0.468 ± 0.090ᵃᵇ | 0.410 ± 0.041ᵃ | 0.639 ± 0.069ᵃᵇ | 0.926 ± 0.130ᵇ | 0.798 ± 0.195ᵃᵇ | 0.533 ± 0.058ᵃᵇ | 0.423 ± 0.063ᵃᵇ | 0.475 ± 0.019ᵃᵇ | 0.537 ± 0.030ᵃᵇ | 0.586 ± 0.062ᵃᵇ | 0.697 ± 0.040ᵃᵇ | 0.743 ± 0.059ᵃᵇ | 0.666 ± 0.009ᵃᵇ | 0.757 ± 0.136ᵃᵇ | 0.626 ± 0.072ᵃᵇ |
| **Naringenin-O-hexoside-4** | 0.131 ± 0.012ᵃ | 0.171 ± 0.034ᵃ | 0.296 ± 0.017ᵃ | 0.249 ± 0.020ᵃ | 0.307 ± 0.027ᵃ | 0.297 ± 0.032ᵃ | 0.349 ± 0.039ᵇ | 0.319 ± 0.044ᵃ | 0.386 ± 0.026ᵃᵇ | 0.318 ± 0.016ᵃᵇ | 0.328 ± 0.020ᵃᵇ | 0.464 ± 0.046ᶜᵈ | 0.499 ± 0.052ᶜᵈ | 0.441 ± 0.047ᶜᵈ | 0.525 ± 0.040ᵈ | 0.492 ± 0.062ᶜᵈ | 0.558 ± 0.092ᵈ | 0.499 ± 0.037ᶜᵈ |

**Table S 6:** Concentration of individual flavonols in haskap pomace samples (mg/kg). Values represent means of four independent repetitions (n = 4). C = control sample; F = frozen sample; P1-P16 = samples treated using pulsed electric field (PEF) under different processing conditions. Mean values followed by different superscript letters within each compound are significantly different according to Tukey’s HSD test (*p* ≤ 0.05).

| **Compound** | **C** | **F** | **P1** | **P2** | **P3** | **P4** | **P5** | **P6** | **P7** | **P8** | **P9** | **P10** | **P11** | **P12** | **P13** | **P14** | **P15** | **P16** |
| --- | --- | --- | --- | --- | --- | --- | --- | --- | --- | --- | --- | --- | --- | --- | --- | --- | --- | --- |
| **Isorhamnetin-3-O-rutinoside** | 8.804 ± 1.866ᵃ | 12.090 ± 3.758ᵃᵇ | 16.632 ± 2.544ᵃᵇ | 14.429 ± 3.022ᵃᵇ | 12.469 ± 2.001ᵃᵇ | 18.505 ± 2.427ᵃᵇ | 21.060 ± 1.839ᵇ | 18.531 ± 2.969ᵃᵇ | 14.219 ± 0.767ᵃᵇ | 13.470 ± 2.204ᵃᵇ | 13.468 ± 0.458ᵃᵇ | 15.807 ± 0.959ᵃᵇ | 18.542 ± 2.061ᵃᵇ | 20.640 ± 1.007ᵇ | 16.788 ± 0.712ᵃᵇ | 16.446 ± 1.229ᵃᵇ | 15.859 ± 1.973ᵃᵇ | 15.624 ± 1.569ᵃᵇ |
| **Isorhamnetin-O-acetyl-rhamnosyl-hexoside** | 0.284 ± 0.024ᵃ | 0.328 ± 0.043ᵃ | 0.581 ± 0.132ᵃᵇ | 0.604 ± 0.123ᵃᵇ | 1.145 ± 0.077ᶜᵈ | 1.053 ± 0.212ᵇᶜᵈ | 1.266 ± 0.141ᶜᵈ | 1.505 ± 0.146ᶜ | 0.756 ± 0.041ᵃᵈ | 0.868 ± 0.090ᵇᵈ | 0.941 ± 0.051ᵇᵈ | 1.241 ± 0.062ᶜᵈ | 1.165 ± 0.058ᶜᵈ | 0.998 ± 0.076ᵇᶜᵈ | 0.970 ± 0.049ᵇᵈ | 1.043 ± 0.108ᵇᶜᵈ | 1.023 ± 0.120ᵇᶜᵈ | 1.008 ± 0.066ᵇᶜᵈ |
| **Isorhamnetin-dihexoside-pentoside** | 0.012 ± 0.003ᵃ | 0.016 ± 0.005ᵃᵇ | 0.020 ± 0.003ᵃᵇ | 0.020 ± 0.004ᵃᵇ | 0.016 ± 0.003ᵃᵇ | 0.023 ± 0.003ᵃᵇ | 0.027 ± 0.004ᵇ | 0.022 ± 0.003ᵃᵇ | 0.022 ± 0.002ᵃᵇ | 0.016 ± 0.003ᵃᵇ | 0.018 ± 0.001ᵃᵇ | 0.018 ± 0.001ᵃᵇ | 0.021 ± 0.002ᵃᵇ | 0.024 ± 0.001ᵃᵇ | 0.020 ± 0.001ᵃᵇ | 0.021 ± 0.001ᵃᵇ | 0.024 ± 0.003ᵃᵇ | 0.022 ± 0.002ᵃᵇ |
| **Isorhamnetin-dirhamnoside-hexoside** | 0.082 ± 0.020ᵃ | 0.105 ± 0.030ᵃᵇ | 0.132 ± 0.021ᵃᵇ | 0.130 ± 0.027ᵃᵇ | 0.107 ± 0.019ᵃᵇ | 0.152 ± 0.021ᵃᵇ | 0.182 ± 0.024ᵇ | 0.148 ± 0.020ᵃᵇ | 0.146 ± 0.016ᵃᵇ | 0.106 ± 0.019ᵃᵇ | 0.123 ± 0.004ᵃᵇ | 0.119 ± 0.009ᵃᵇ | 0.139 ± 0.011ᵃᵇ | 0.163 ± 0.009ᵃᵇ | 0.131 ± 0.003ᵃᵇ | 0.141 ± 0.007ᵃᵇ | 0.161 ± 0.022ᵃᵇ | 0.148 ± 0.013ᵃᵇ |
| **Isorhamnetin-O-hexoside** | 0.244 ± 0.076ᵃ | 0.245 ± 0.077ᵃ | 0.347 ± 0.044ᵃᵇ | 0.298 ± 0.076ᵃ | 0.318 ± 0.035ᵃᶠ | 0.352 ± 0.031ᵃᵇ | 0.485 ± 0.044ᵃᵇᵉ | 0.386 ± 0.065ᵃᵇᶜ | 0.576 ± 0.031ᵇᵉᶠ | 0.478 ± 0.039ᵃᵇᵉ | 0.359 ± 0.034ᵃᵇ | 0.394 ± 0.019ᵃᵇᶜ | 0.451 ± 0.030ᵃᵇᶜᵈ | 0.590 ± 0.075ᵇᵉ | 0.681 ± 0.028ᵈᵉ | 0.734 ± 0.058ᵉ | 0.646 ± 0.049ᶜᵉ | 0.723 ± 0.056ᵉ |
| **Isorhamnetin-O-hexoside-pentoside** | 0.602 ± 0.085ᵃ | 0.861 ± 0.203ᵃᵇ | 1.111 ± 0.159ᵃᵇᶜ | 0.794 ± 0.181ᵃᵇ | 0.727 ± 0.114ᵃᶠ | 1.079 ± 0.140ᵃᵇᶜ | 1.217 ± 0.136ᵃᵇᶜ | 1.101 ± 0.183ᵃᵇᶜ | 0.879 ± 0.047ᵃᵇᵉ | 0.860 ± 0.121ᵃᵇ | 0.994 ± 0.045ᵃᵇᵈ | 1.751 ± 0.150ᶜᵈ | 1.713 ± 0.236ᶜᵈᵉ | 1.899 ± 0.171ᶜ | 1.585 ± 0.170ᵇᶜ | 1.219 ± 0.228ᵃᵇᶜ | 1.495 ± 0.239ᵇᶜᶠ | 1.554 ± 0.111ᵇᶜᶠ |
| **Kaempferol-3-O-galactoside** | 0.045 ± 0.007ᵃ | 0.063 ± 0.019ᵃᵇ | 0.092 ± 0.014ᵃᶜᵈ | 0.078 ± 0.017ᵃᵈ | 0.096 ± 0.017ᵃᶜᵈ | 0.122 ± 0.017ᵇᵈᵉ | 0.125 ± 0.010ᵇᵈᵉ | 0.116 ± 0.018ᵇᵈᵉ | 0.098 ± 0.006ᵃᶜᵈ | 0.095 ± 0.010ᵃᶜᵈ | 0.104 ± 0.003ᵃᵉ | 0.138 ± 0.006ᵈᵉ | 0.153 ± 0.019ᶜᵉ | 0.166 ± 0.009ᵉ | 0.132 ± 0.002ᵈᵉ | 0.139 ± 0.011ᵈᵉ | 0.126 ± 0.006ᵇᵈᵉ | 0.120 ± 0.009ᵇᵈᵉ |
| **Kaempferol-3-O-glucoside** | 0.365 ± 0.103ᵃ | 0.467 ± 0.120ᵃ | 0.736 ± 0.063ᵃᵇᶜ | 0.634 ± 0.135ᵃᶜ | 0.790 ± 0.162ᵃᵇᶜ | 0.408 ± 0.047ᵃ | 0.960 ± 0.117ᵃᵈ | 0.514 ± 0.102ᵃ | 0.844 ± 0.042ᵃᵇᶜ | 0.718 ± 0.038ᵃᵇᶜ | 0.605 ± 0.049ᵃᶜ | 0.824 ± 0.076ᵃᵇᶜ | 1.200 ± 0.114ᶜᵈ | 2.006 ± 0.231ᵉ | 1.593 ± 0.229ᵈᵉ | 1.336 ± 0.168ᵇᵈ | 1.531 ± 0.122ᵈᵉ | 1.524 ± 0.083ᵈᵉ |
| **Kaempferol-3-O-rutinoside** | 1.076 ± 0.253ᵃ | 1.407 ± 0.374ᵃᵇ | 1.741 ± 0.249ᵃᶜ | 1.698 ± 0.386ᵃᶜ | 1.284 ± 0.225ᵃᵇ | 1.940 ± 0.291ᵃᶜ | 2.568 ± 0.252ᵇᶜᵈ | 2.105 ± 0.241ᵃᶜᵉ | 2.349 ± 0.176ᵃᵈ | 1.866 ± 0.221ᵃᶜ | 1.892 ± 0.060ᵃᶜ | 2.250 ± 0.333ᵃᵈ | 2.301 ± 0.194ᵃᵈ | 2.005 ± 0.097ᵃᶜ | 2.849 ± 0.232ᶜᵈ | 3.561 ± 0.439ᵈ | 3.453 ± 0.314ᵈᵉ | 2.883 ± 0.205ᶜᵈ |
| **Kaempferol-O-acetyl-hexoside** | 0.058 ± 0.003ᵃ | 0.058 ± 0.008ᵃ | 0.077 ± 0.010ᵃᵇ | 0.059 ± 0.008ᵃ | 0.086 ± 0.011ᵃᵈ | 0.077 ± 0.009ᵃᵇ | 0.109 ± 0.027ᵃᵉ | 0.088 ± 0.010ᵃᵈ | 0.100 ± 0.004ᵃᵉ | 0.129 ± 0.005ᵃᵉᶠ | 0.137 ± 0.006ᵇᵈᵉᶠ | 0.160 ± 0.022ᶜᵈᵉ | 0.172 ± 0.012ᶜᵉ | 0.192 ± 0.024ᶜᶠ | 0.289 ± 0.012ᵍʰ | 0.220 ± 0.018ᶜᵍ | 0.300 ± 0.029ʰ | 0.269 ± 0.008ᵍʰ |
| **Kaempferol-O-hexoside-pentoside** | 0.048 ± 0.010ᵃ | 0.066 ± 0.015ᵃᵇ | 0.075 ± 0.013ᵃᶜ | 0.110 ± 0.024ᵃᶠ | 0.098 ± 0.008ᵃᶠ | 0.115 ± 0.013ᵃᶠ | 0.141 ± 0.002ᵇᶜᵉᶠ | 0.123 ± 0.012ᵃᶠᵍ | 0.164 ± 0.011ᵉʰ | 0.126 ± 0.007ᵃᵉᶠ | 0.149 ± 0.001ᶜᵉᶠ | 0.179 ± 0.005ᵈᵉᶠ | 0.198 ± 0.009ᵈᵉᵍ | 0.206 ± 0.006ᵈᵉ | 0.233 ± 0.020ᵈʰ | 0.250 ± 0.044ᵈ | 0.244 ± 0.017ᵈʰ | 0.167 ± 0.012ᵉʰ |
| **Quercetin-O-glycoside** | 1.350 ± 0.380ᵃᵇ | 1.711 ± 0.498ᵃᵇ | 1.955 ± 0.121ᵃᵇ | 1.658 ± 0.352ᵃᵇ | 1.497 ± 0.255ᵃᵇ | 2.206 ± 0.308ᵃ | 2.246 ± 0.194ᵃ | 2.024 ± 0.221ᵃᵇ | 0.894 ± 0.024ᵇ | 1.086 ± 0.190ᵃᵇ | 1.029 ± 0.065ᵇ | 1.210 ± 0.134ᵃᵇ | 1.403 ± 0.154ᵃᵇ | 1.585 ± 0.081ᵃᵇ | 1.537 ± 0.043ᵃᵇ | 1.359 ± 0.078ᵃᵇ | 1.298 ± 0.137ᵃᵇ | 1.365 ± 0.129ᵃᵇ |
| **Quercetin-3-O-arabinofuranoside** | 0.014 ± 0.004ᵃ | 0.019 ± 0.005ᵃ | 0.029 ± 0.003ᵃᵇᶜ | 0.025 ± 0.005ᵃᶜ | 0.031 ± 0.006ᵃᵇᶜ | 0.016 ± 0.002ᵃ | 0.038 ± 0.005ᵃᵈ | 0.020 ± 0.004ᵃ | 0.033 ± 0.002ᵃᵇᶜ | 0.028 ± 0.002ᵃᵇᶜ | 0.024 ± 0.002ᵃᶜ | 0.033 ± 0.003ᵃᵇᶜ | 0.047 ± 0.005ᶜᵈ | 0.079 ± 0.009ᵉ | 0.063 ± 0.009ᵈᵉ | 0.053 ± 0.007ᵇᵈ | 0.061 ± 0.005ᵈᵉ | 0.060 ± 0.003ᵈᵉ |
| **Quercetin-3-O-arabinopyranoside** | 0.590 ± 0.097ᵃ | 0.831 ± 0.245ᵃᵇ | 1.208 ± 0.177ᵃᶜᵈ | 1.025 ± 0.219ᵃᵈ | 1.254 ± 0.220ᵃᶜᵈ | 1.604 ± 0.226ᵇᵈᵉ | 1.647 ± 0.137ᵇᵈᵉ | 1.525 ± 0.241ᵇᵈᵉ | 1.289 ± 0.081ᵃᶜᵈ | 1.245 ± 0.132ᵃᶜᵈ | 1.362 ± 0.039ᵃᵉ | 1.817 ± 0.074ᵈᵉ | 2.009 ± 0.243ᶜᵉ | 2.180 ± 0.117ᵉ | 1.729 ± 0.027ᵈᵉ | 1.826 ± 0.146ᵈᵉ | 1.651 ± 0.080ᵇᵈᵉ | 1.569 ± 0.112ᵇᵈᵉ |
| **Quercetin-3-O-galactoside** | 7.490 ± 1.599ᵃ | 10.047 ± 3.128ᵃᵇ | 13.948 ± 2.145ᵃᵇ | 12.474 ± 2.651ᵃᵇ | 10.760 ± 1.668ᵃᵇ | 15.336 ± 1.907ᵃᵇ | 17.132 ± 1.489ᵃᵇ | 15.119 ± 2.763ᵃᵇ | 12.378 ± 0.572ᵃᵇ | 11.437 ± 1.800ᵃᵇ | 11.873 ± 0.329ᵃᵇ | 13.853 ± 0.795ᵃᵇ | 16.348 ± 1.638ᵃᵇ | 18.213 ± 0.859ᵇ | 13.703 ± 0.718ᵃᵇ | 13.511 ± 1.013ᵃᵇ | 12.918 ± 1.714ᵃᵇ | 12.596 ± 1.265ᵃᵇ |
| **Quercetin-3-O-glucoside** | 0.282 ± 0.057ᵃ | 0.392 ± 0.088ᵃᵇ | 0.443 ± 0.078ᵃᶜ | 0.653 ± 0.145ᵃᶠ | 0.580 ± 0.045ᵃᶠ | 0.682 ± 0.075ᵃᶠ | 0.833 ± 0.014ᵇᶜᵉᶠ | 0.729 ± 0.070ᵃᶠᵍ | 0.972 ± 0.065ᵉʰ | 0.749 ± 0.042ᵃᵉᶠ | 0.881 ± 0.004ᶜᵉᶠ | 1.060 ± 0.032ᵈᵉᶠ | 1.175 ± 0.053ᵈᵉᵍ | 1.225 ± 0.035ᵈᵉ | 1.380 ± 0.119ᵈʰ | 1.482 ± 0.262ᵈ | 1.450 ± 0.101ᵈʰ | 0.988 ± 0.074ᵉʰ |
| **Quercetin-3-O-rhamnoside** | 0.046 ± 0.013ᵃ | 0.058 ± 0.015ᵃ | 0.092 ± 0.008ᵃᵇᶜ | 0.079 ± 0.017ᵃᶜ | 0.098 ± 0.020ᵃᵇᶜ | 0.051 ± 0.006ᵃ | 0.120 ± 0.015ᵃᵈ | 0.064 ± 0.013ᵃ | 0.105 ± 0.005ᵃᵇᶜ | 0.089 ± 0.005ᵃᵇᶜ | 0.075 ± 0.006ᵃᶜ | 0.103 ± 0.010ᵃᵇᶜ | 0.149 ± 0.014ᶜᵈ | 0.250 ± 0.029ᵉ | 0.198 ± 0.029ᵈᵉ | 0.166 ± 0.021ᵇᵈ | 0.191 ± 0.015ᵈᵉ | 0.190 ± 0.010ᵈᵉ |
| **Quercetin-3-O-rutinoside** | 6.299 ± 1.493ᵃ | 7.707 ± 2.231ᵃᵇ | 9.459 ± 1.428ᵃᵇ | 9.227 ± 1.786ᵃᵇ | 7.723 ± 1.423ᵃᵇ | 10.999 ± 1.515ᵃᵇ | 13.660 ± 1.730ᵇ | 11.133 ± 1.506ᵃᵇ | 10.764 ± 1.207ᵃᵇ | 8.143 ± 1.368ᵃᵇ | 9.079 ± 0.344ᵃᵇ | 8.239 ± 0.564ᵃᵇ | 9.982 ± 0.784ᵃᵇ | 11.818 ± 0.745ᵃᵇ | 9.195 ± 0.309ᵃᵇ | 9.962 ± 0.621ᵃᵇ | 11.556 ± 1.860ᵃᵇ | 10.850 ± 1.021ᵃᵇ |
| **Quercetin-3-O-xyloside** | 0.982 ± 0.139ᵃ | 1.405 ± 0.332ᵃᵇᶜ | 1.768 ± 0.289ᵃᶜᵈᵉ | 1.296 ± 0.296ᵃᶜ | 1.187 ± 0.186ᵃ | 1.799 ± 0.246ᵃᶜᵈᵉ | 2.247 ± 0.281ᵃᵇᶜᶠ | 1.937 ± 0.182ᵃᵇᶜᶠ | 1.368 ± 0.050ᵃᵇᶜ | 1.404 ± 0.197ᵃᵇᶜ | 1.622 ± 0.073ᵃᶜᵉ | 2.858 ± 0.245ᵉᶠ | 2.660 ± 0.271ᵇᵉᶠ | 2.541 ± 0.468ᵇᶜᶠ | 2.254 ± 0.099ᵃᵇᶜᶠ | 3.132 ± 0.386ᶠ | 3.037 ± 0.276ᵈᶠ | 2.536 ± 0.180ᵇᶜᶠ |
| **Quercetin-O-acetyl-hexoside** | 4.281 ± 0.967ᵃᵇ | 4.387 ± 1.737ᵃᵇ | 4.719 ± 0.575ᵃᵇ | 3.809 ± 0.728ᵃᵇ | 3.335 ± 0.331ᵃ | 5.195 ± 0.561ᵃᵇ | 7.533 ± 1.058ᵇ | 6.493 ± 1.584ᵃᵇ | 4.335 ± 0.474ᵃᵇ | 3.441 ± 0.509ᵃᵇ | 3.862 ± 0.157ᵃᵇ | 4.368 ± 0.244ᵃᵇ | 4.765 ± 0.506ᵃᵇ | 5.669 ± 0.321ᵃᵇ | 6.046 ± 0.482ᵃᵇ | 5.418 ± 0.074ᵃᵇ | 6.158 ± 1.107ᵃᵇ | 5.094 ± 0.584ᵃᵇ |
| **Quercetin-O-hexoside** | 0.300 ± 0.062ᵃ | 0.510 ± 0.114ᵃᵇ | 0.619 ± 0.083ᵃᵇ | 0.504 ± 0.111ᵃ | 0.448 ± 0.065ᵃ | 0.631 ± 0.081ᵃᵇ | 0.701 ± 0.039ᵃᵈ | 0.693 ± 0.119ᵃᵈ | 0.605 ± 0.066ᵃᵇ | 0.546 ± 0.039ᵃᵇ | 0.896 ± 0.131ᵃᵈᵉ | 1.279 ± 0.066ᶜᵈ | 1.136 ± 0.107ᵇᶜᵈ | 1.149 ± 0.139ᵇᶜᵈ | 1.644 ± 0.123ᶜ | 1.131 ± 0.175ᵇᶜᵈ | 1.327 ± 0.267ᶜᵉ | 1.617 ± 0.098ᶜ |
| **Quercetin-O-hexoside-pentoside** | 0.070 ± 0.016ᵃ | 0.249 ± 0.063ᵃᵇ | 0.321 ± 0.045ᵇ | 0.303 ± 0.058ᵇ | 0.251 ± 0.035ᵃᵇ | 0.374 ± 0.048ᵇ | 0.349 ± 0.028ᵇ | 0.320 ± 0.043ᵇ | 0.240 ± 0.017ᵃᵇ | 0.194 ± 0.035ᵃᵇ | 0.222 ± 0.015ᵃᵇ | 0.221 ± 0.023ᵃᵇ | 0.269 ± 0.027ᵇ | 0.296 ± 0.014ᵇ | 0.245 ± 0.018ᵃᵇ | 0.238 ± 0.007ᵃᵇ | 0.313 ± 0.046ᵇ | 0.299 ± 0.038ᵇ |
| **Quercetin-O-vicianoside** | 3.080 ± 0.739ᵃ | 3.915 ± 1.116ᵃᵇ | 4.926 ± 0.768ᵃᵇ | 4.868 ± 0.994ᵃᵇ | 4.020 ± 0.719ᵃᵇ | 5.689 ± 0.792ᵃᵇ | 6.824 ± 0.887ᵇ | 5.557 ± 0.743ᵃᵇ | 5.460 ± 0.598ᵃᵇ | 3.958 ± 0.698ᵃᵇ | 4.594 ± 0.133ᵃᵇ | 4.464 ± 0.354ᵃᵇ | 5.224 ± 0.414ᵃᵇ | 6.101 ± 0.322ᵃᵇ | 4.896 ± 0.128ᵃᵇ | 5.274 ± 0.247ᵃᵇ | 6.016 ± 0.829ᵃᵇ | 5.545 ± 0.480ᵃᵇ |

**Table S 7:** Concentration of individual flavanols in haskap pomace samples (mg/kg). Values represent means of four independent repetitions (n = 4). C = control sample; F = frozen sample; P1-P16 = samples treated using pulsed electric field (PEF) under different processing conditions. Mean values followed by different superscript letters within each compound are significantly different according to Tukey’s HSD test (*p* ≤ 0.05).

| **Compound** | **C** | **F** | **P1** | **P2** | **P3** | **P4** | **P5** | **P6** | **P7** | **P8** | **P9** | **P10** | **P11** | **P12** | **P13** | **P14** | **P15** | **P16** |
| --- | --- | --- | --- | --- | --- | --- | --- | --- | --- | --- | --- | --- | --- | --- | --- | --- | --- | --- |
| **Catechin** | 2.09 ± 0.67ᵃ | 2.20 ± 0.65ᵃᵇ | 2.78 ± 0.42ᵃᵇ | 2.74 ± 0.53ᵃᵇ | 2.19 ± 0.51ᵃᵇ | 3.07 ± 0.45ᵃᵇ | 4.24 ± 0.46ᵇ | 3.29 ± 0.44ᵃᵇ | 3.48 ± 0.44ᵃᵇ | 2.66 ± 0.40ᵃᵇ | 2.70 ± 0.07ᵃᵇ | 2.40 ± 0.13ᵃᵇ | 3.25 ± 0.25ᵃᵇ | 3.40 ± 0.20ᵃᵇ | 2.97 ± 0.10ᵃᵇ | 3.01 ± 0.14ᵃᵇ | 3.25 ± 0.52ᵃᵇ | 3.21 ± 0.30ᵃᵇ |
| **Epicatechin** | 14.01 ± 4.21ᵃ | 20.06 ± 4.14ᵃᵇ | 21.80 ± 3.37ᵃᵇ | 23.71 ± 4.18ᵃᵇ | 21.01 ± 4.11ᵃᵇ | 21.11 ± 5.36ᵃᵇ | 35.73 ± 1.37ᵇ | 31.50 ± 4.45ᵇ | 22.48 ± 1.94ᵃᵇ | 19.30 ± 1.88ᵃᵇ | 23.18 ± 0.96ᵃᵇ | 28.19 ± 0.77ᵃᵇ | 31.83 ± 2.52ᵇ | 32.40 ± 1.53ᵇ | 33.68 ± 2.75ᵇ | 29.09 ± 1.68ᵃᵇ | 34.66 ± 3.06ᵇ | 30.78 ± 4.58ᵃᵇ |
| **Procyanidin dimer 1** | 21.59 ± 2.32ᵃ | 24.46 ± 6.28ᵃ | 59.35 ± 3.65ᵃᵇ | 42.92 ± 7.12ᵃᶜ | 43.67 ± 6.99ᵃᶜ | 51.35 ± 7.26ᵃᵇ | 97.42 ± 23.05ᵇᵈ | 83.91 ± 11.91ᵇᶜᵈ | 61.86 ± 7.35ᵃᵇ | 66.17 ± 14.33ᵃᵇᵉ | 67.06 ± 3.59ᵃᵇᵉ | 72.90 ± 1.28ᵇᶜᵈ | 84.47 ± 11.41ᵇᶜᵈ | 87.44 ± 8.43ᵇᶜᵈ | 111.38 ± 3.13ᵈᵉ | 108.83 ± 5.46ᵈᵉ | 114.44 ± 6.50ᵈ | 95.50 ± 6.33ᵇᵈ |
| **Procyanidin dimer 2** | 8.45 ± 3.76ᵃ | 18.81 ± 3.18ᵃᵇ | 20.25 ± 3.32ᵃᵇᶜ | 20.67 ± 3.69ᵃᵇᶠ | 13.62 ± 2.64ᵃᵈ | 21.34 ± 3.59ᵃᵇᶠ | 28.43 ± 2.83ᵇᵉ | 25.53 ± 3.50ᵇᵈᶠ | 25.89 ± 2.33ᵇᵈᵉᶠ | 21.62 ± 3.34ᵃᵇᶠ | 23.77 ± 0.35ᵇᵈᶠ | 26.48 ± 0.85ᵇᵈᵉᶠ | 30.28 ± 2.18ᵇᵉ | 32.39 ± 1.92ᵇᵉ | 40.44 ± 1.63ᵉ | 33.44 ± 1.52ᵇᵉ | 35.24 ± 4.39ᵉᶠ | 34.80 ± 2.15ᶜᵉᶠ |
| **Procyanidin dimer 3** | 26.94 ± 6.59ᵃ | 36.38 ± 12.88ᵃᵇ | 41.57 ± 10.38ᵃᵇ | 48.19 ± 11.71ᵃᵉ | 45.76 ± 8.67ᵃᵉ | 65.87 ± 9.08ᵃᵈᵉᵍ | 86.72 ± 6.39ᶜᵈᵉ | 72.79 ± 12.02ᵇᵈᵉᵍ | 49.74 ± 4.95ᵃᵉᶠ | 54.31 ± 11.06ᵃᵈ | 67.07 ± 2.15ᵃᵈᵉᵍ | 79.81 ± 1.89ᵇᶜᵈᵉ | 87.66 ± 8.01ᶜᵈᵉ | 92.50 ± 8.31ᶜᵈᶠ | 117.01 ± 4.08ᶜ | 107.20 ± 3.86ᶜᵍ | 118.12 ± 11.23ᶜ | 94.88 ± 7.64ᶜᵈ |
| **Procyanidin dimer 4** | 21.25 ± 7.01ᵃ | 34.22 ± 7.90ᵃᵇ | 34.17 ± 4.20ᵃᵇ | 28.94 ± 4.49ᵃᶜ | 26.24 ± 6.05ᵃᶜ | 40.66 ± 4.80ᵃᵇ | 50.84 ± 3.42ᵇ | 38.93 ± 4.48ᵃᵇ | 34.79 ± 2.16ᵃᵇ | 31.37 ± 4.32ᵃᵇ | 35.15 ± 0.56ᵃᵇ | 35.29 ± 2.11ᵃᵇ | 39.95 ± 2.30ᵃᵇ | 38.70 ± 1.61ᵃᵇ | 43.70 ± 1.04ᵇᶜ | 37.48 ± 2.71ᵃᵇ | 45.41 ± 4.05ᵇᶜ | 41.63 ± 1.41ᵃᵇ |
| **Procyanidin trimer 1** | 1.63 ± 0.52ᵃ | 1.87 ± 0.32ᵃᵇ | 2.24 ± 0.50ᵃᶜ | 2.07 ± 0.64ᵃᵍ | 2.13 ± 0.72ᵃᵈᵍ | 4.03 ± 0.91ᵃᶜᶠ | 5.10 ± 0.86ᵇᶜᵉᶠᵍ | 5.49 ± 0.59ᶜᵉ | 4.32 ± 0.20ᵃᶜᶠ | 5.14 ± 0.75ᶜᵉᶠᵍ | 5.66 ± 0.49ᵉᶠ | 5.36 ± 0.30ᶜᵈᵉ | 5.79 ± 0.45ᵉᶠ | 6.01 ± 0.19ᵉᶠ | 8.09 ± 0.61ᵉ | 7.81 ± 0.96ᵉ | 8.33 ± 0.83ᵉ | 7.82 ± 0.73ᵉ |
| **Procyanidin trimer 2** | 2.93 ± 0.95ᵃ | 3.09 ± 0.92ᵃᵇ | 3.91 ± 0.59ᵃᵇ | 3.84 ± 0.75ᵃᵇ | 3.08 ± 0.72ᵃᵇ | 4.32 ± 0.64ᵃᵇ | 5.97 ± 0.65ᵇ | 4.62 ± 0.62ᵃᵇ | 4.89 ± 0.62ᵃᵇ | 3.74 ± 0.56ᵃᵇ | 3.80 ± 0.10ᵃᵇ | 3.37 ± 0.18ᵃᵇ | 4.56 ± 0.35ᵃᵇ | 4.78 ± 0.28ᵃᵇ | 4.18 ± 0.15ᵃᵇ | 4.23 ± 0.19ᵃᵇ | 4.57 ± 0.73ᵃᵇ | 4.51 ± 0.43ᵃᵇ |
| **Procyanidin trimer 3** | 0.26 ± 0.05ᵃ | 0.42 ± 0.07ᵃᵇ | 0.31 ± 0.07ᵃᶜ | 0.38 ± 0.08ᵃᵇ | 0.33 ± 0.06ᵃᵈ | 0.59 ± 0.08ᵃᵇ | 0.59 ± 0.07ᵃᵇ | 0.49 ± 0.07ᵃᵇ | 0.45 ± 0.05ᵃᵇ | 0.35 ± 0.07ᵃᵇ | 0.43 ± 0.02ᵃᵇ | 2.40 ± 0.13ᵃᵇ | 3.25 ± 0.25ᵃᵇ | 3.40 ± 0.20ᵃᵇ | 2.97 ± 0.10ᵃᵇ | 3.01 ± 0.14ᵃᵇ | 3.25 ± 0.52ᵃᵇ | 3.21 ± 0.30ᵃᵇ |
| **Procyanidin trimer 4** | 0.54 ± 0.13ᵃ | 0.73 ± 0.26ᵃᵇ | 0.83 ± 0.21ᵃᵇ | 0.96 ± 0.23ᵃᵉ | 0.92 ± 0.17ᵃᵉ | 1.32 ± 0.18ᵃᵈᵉᵍ | 1.73 ± 0.13ᶜᵈᵉ | 1.46 ± 0.24ᵇᵈᵉᵍ | 0.99 ± 0.10ᵃᵉᶠ | 1.09 ± 0.22ᵃᵈ | 1.34 ± 0.04ᵃᵈᵉᵍ | 28.19 ± 0.77ᵃᵇ | 31.83 ± 2.52ᵇ | 32.40 ± 1.53ᵇ | 33.68 ± 2.75ᵇ | 29.09 ± 1.68ᵃᵇ | 34.66 ± 3.06ᵇ | 30.78 ± 4.58ᵃᵇ |
| **Procyanidin trimer 5** | 0.51 ± 0.17ᵃ | 0.82 ± 0.19ᵃᵇ | 0.82 ± 0.10ᵃᵇ | 0.69 ± 0.11ᵃᶜ | 0.63 ± 0.15ᵃᶜ | 0.98 ± 0.12ᵃᵇ | 1.22 ± 0.08ᵇ | 0.93 ± 0.11ᵃᵇ | 0.83 ± 0.05ᵃᵇ | 0.75 ± 0.10ᵃᵇ | 0.84 ± 0.01ᵃᵇ | 72.90 ± 1.28ᵇᶜᵈ | 84.47 ± 11.41ᵇᶜᵈ | 87.44 ± 8.43ᵇᶜᵈ | 111.38 ± 3.13ᵈᵉ | 108.83 ± 5.46ᵈᵉ | 114.44 ± 6.50ᵈ | 95.50 ± 6.33ᵇᵈ |
| **Procyanidin trimer 6** | 12.04 ± 3.75ᵃ | 20.55 ± 5.10ᵃᵇ | 20.50 ± 2.03ᵃᵇ | 16.43 ± 2.13ᵃᵇ | 11.99 ± 1.89ᵃ | 16.02 ± 2.40ᵃᵇ | 18.39 ± 2.71ᵃᵇ | 19.03 ± 2.15ᵃᵇ | 20.05 ± 1.97ᵃᵇ | 15.08 ± 2.41ᵃᵇ | 17.58 ± 0.80ᵃᵇ | 26.48 ± 0.85ᵇᵈᵉᶠ | 30.28 ± 2.18ᵇᵉ | 32.39 ± 1.92ᵇᵉ | 40.44 ± 1.63ᵉ | 33.44 ± 1.52ᵇᵉ | 35.24 ± 4.39ᵉᶠ | 34.80 ± 2.15ᶜᵉᶠ |

**Table S 8:** Concentration of individual hydroxycinnamic acids in haskap pomace samples (mg/kg). Values represent means of four independent repetitions (n = 4). C = control sample; F = frozen sample; P1-P16 = samples treated using pulsed electric field (PEF) under different processing conditions. Mean values followed by different superscript letters within each compound are significantly different according to Tukey’s HSD test (*p* ≤ 0.05).

| **Compound** | **C** | **F** | **P1** | **P2** | **P3** | **P4** | **P5** | **P6** | **P7** | **P8** | **P9** | **P10** | **P11** | **P12** | **P13** | **P14** | **P15** | **P16** |
| --- | --- | --- | --- | --- | --- | --- | --- | --- | --- | --- | --- | --- | --- | --- | --- | --- | --- | --- |
| **Caffeic acid** | 0.25 ± 0.08ᵃ | 0.36 ± 0.07ᵃᵇ | 0.39 ± 0.06ᵃᵇ | 0.43 ± 0.08ᵃᵇ | 0.38 ± 0.07ᵃᵇ | 0.38 ± 0.10ᵃᵇ | 0.65 ± 0.02ᵇ | 0.57 ± 0.08ᵇ | 0.41 ± 0.04ᵃᵇ | 0.35 ± 0.03ᵃᵇ | 0.42 ± 0.02ᵃᵇ | 0.51 ± 0.01ᵃᵇ | 0.58 ± 0.05ᵇ | 0.59 ± 0.03ᵇ | 0.61 ± 0.05ᵇ | 0.53 ± 0.03ᵃᵇ | 0.63 ± 0.06ᵇ | 0.56 ± 0.08ᵃᵇ |
| **Caffeic acid-O-hexoside** | 1.08 ± 0.48ᵃ | 2.40 ± 0.40ᵃᵇ | 2.58 ± 0.42ᵃᵇᶜ | 2.64 ± 0.47ᵃᵇᶠ | 1.74 ± 0.34ᵃᵈ | 2.72 ± 0.46ᵃᵇᶠ | 3.62 ± 0.36ᵇᵉ | 3.25 ± 0.45ᵇᵈᶠ | 3.30 ± 0.30ᵇᵈᵉᶠ | 2.76 ± 0.43ᵃᵇᶠ | 3.03 ± 0.04ᵇᵈᶠ | 3.38 ± 0.11ᵇᵈᵉᶠ | 3.86 ± 0.28ᵇᵉ | 4.13 ± 0.25ᵇᵉ | 5.15 ± 0.21ᵉ | 4.26 ± 0.19ᶜᵉᶠ | 4.49 ± 0.56ᵉᶠ | 4.44 ± 0.27ᶜᵉᶠ |
| **3,5-Dicaffeoylquinic acid (or Dicaffeoylquinic acid I)** | 0.98 ± 0.22ᵃᵇ | 1.00 ± 0.40ᵃᵇ | 1.08 ± 0.13ᵃᵇ | 0.87 ± 0.17ᵃᵇ | 0.76 ± 0.08ᵃ | 1.18 ± 0.13ᵃᵇ | 1.72 ± 0.24ᵇ | 1.48 ± 0.36ᵃᵇ | 0.99 ± 0.11ᵃᵇ | 0.78 ± 0.12ᵃᵇ | 0.88 ± 0.04ᵃᵇ | 1.00 ± 0.06ᵃᵇ | 1.09 ± 0.12ᵃᵇ | 1.29 ± 0.07ᵃᵇ | 1.38 ± 0.11ᵃᵇ | 1.23 ± 0.02ᵃᵇ | 1.40 ± 0.25ᵃᵇ | 1.16 ± 0.13ᵃᵇ |
| **3,4-Dicaffeoylquinic acid (or Dicaffeoylquinic acid II)** | 0.23 ± 0.04ᵃᵇ | 0.21 ± 0.03ᵇ | 0.34 ± 0.01ᵇᶜ | 0.35 ± 0.01ᵇᶜ | 0.37 ± 0.04ᵇᶜᶠ | 0.46 ± 0.04ᶜᵉ | 0.55 ± 0.07ᶜᵍ | 0.59 ± 0.08ᵉᵍ | 0.46 ± 0.02ᶜᵉ | 0.44 ± 0.03ᵃᶜᵉ | 0.35 ± 0.01ᵇᶜ | 0.51 ± 0.04ᶜᵈᵉ | 0.53 ± 0.05ᶜᵈᵉ | 0.56 ± 0.03ᵉᶠᵍ | 0.70 ± 0.03ᵈᵍ | 0.67 ± 0.06ᵈᵍ | 0.70 ± 0.04ᵈᵍ | 0.74 ± 0.01ᵍ |
| **p-Coumaric acid-O-hexoside** | 1.11 ± 0.24ᵃᵇ | 0.70 ± 0.11ᵇ | 0.77 ± 0.03ᵇ | 1.12 ± 0.20ᵃᵇ | 0.81 ± 0.08ᵇ | 0.95 ± 0.09ᵇᵈ | 1.50 ± 0.18ᵃᵇ | 1.24 ± 0.25ᵃᵇ | 1.04 ± 0.26ᵇᶜᵈ | 0.96 ± 0.09ᵇᵈ | 1.14 ± 0.10ᵃᵇ | 1.01 ± 0.13ᵇᶜᵈ | 1.35 ± 0.14ᵃᵇ | 1.34 ± 0.20ᵃᵇ | 1.39 ± 0.17ᵃᵇ | 1.76 ± 0.08ᵃᵈ | 1.85 ± 0.13ᵃᶜ | 1.93 ± 0.24ᵃ |
| **3-O-Caffeoylquinic acid** | 54.67 ± 8.30ᵃ | 79.29 ± 20.40ᵃᵇ | 111.81 ± 14.05ᵃᶜ | 81.61 ± 17.53ᵃᶜ | 79.79 ± 13.51ᵃᵇ | 110.51 ± 13.40ᵃᶜ | 141.23 ± 8.86ᶜ | 113.86 ± 18.42ᵃᶜ | 67.70 ± 6.87ᵃ | 77.23 ± 12.37ᵃᵇ | 97.89 ± 3.72ᵃᶜ | 103.35 ± 3.99ᵃᶜ | 107.18 ± 5.34ᵃᶜ | 106.00 ± 4.79ᵃᶜ | 128.86 ± 8.23ᵇᶜ | 111.19 ± 6.54ᵃᶜ | 108.19 ± 14.21ᵃᶜ | 106.36 ± 5.62ᵃᶜ |
| **3-O-Feruloylquinic acid** | 0.00 ± 0.00ᵃ | 0.01 ± 0.00ᵃᵇ | 0.00 ± 0.00ᵃᶜ | 0.01 ± 0.00ᵃᵇ | 0.00 ± 0.00ᵃᵈ | 0.01 ± 0.00ᵃᵇ | 0.01 ± 0.00ᵃᵇ | 0.01 ± 0.00ᵃᵇ | 0.01 ± 0.00ᵃᵇ | 0.00 ± 0.00ᵃᵇ | 0.01 ± 0.00ᵃᵇ | 0.01 ± 0.00ᵃᵇ | 0.01 ± 0.00ᵃᵇ | 0.01 ± 0.00ᵇᵈ | 0.01 ± 0.00ᵇᶜᵈ | 0.01 ± 0.00ᵇ | 0.01 ± 0.00ᵇᶜᵈ | 0.01 ± 0.00ᵇᶜᵈ |
| **3-O-p-Coumaroylquinic acid** | 0.78 ± 0.25ᵃ | 0.90 ± 0.15ᵃᵇ | 1.08 ± 0.24ᵃᶜ | 1.00 ± 0.31ᵃᵍ | 1.03 ± 0.34ᵃᵈᵍ | 1.94 ± 0.44ᵃᶜᶠ | 2.45 ± 0.41ᵇᶜᵉᶠᵍ | 2.64 ± 0.29ᶜᵉ | 2.08 ± 0.10ᵃᶜᶠ | 2.47 ± 0.36ᶜᵉᶠᵍ | 2.72 ± 0.23ᵉᶠ | 2.58 ± 0.15ᶜᵈᵉ | 2.78 ± 0.22ᵉᶠ | 2.89 ± 0.09ᵉᶠ | 3.89 ± 0.29ᵉ | 3.76 ± 0.46ᵉ | 4.00 ± 0.40ᵉ | 3.76 ± 0.35ᵉ |
| **4-O-Caffeoylquinic acid** | 2.61 ± 0.49ᵃ | 4.21 ± 0.72ᵃᵇ | 3.12 ± 0.72ᵃᶜ | 3.76 ± 0.79ᵃᵇ | 3.28 ± 0.56ᵃᵈ | 5.88 ± 0.78ᵃᵇ | 5.91 ± 0.71ᵃᵇ | 4.86 ± 0.69ᵃᵇ | 4.51 ± 0.53ᵃᵇ | 3.51 ± 0.71ᵃᵇ | 4.29 ± 0.18ᵃᵇ | 4.62 ± 0.36ᵃᵇ | 5.24 ± 0.67ᵃᵇ | 5.61 ± 0.30ᵃᵇ | 6.60 ± 1.34ᵇᵈ | 6.21 ± 0.33ᵇᶜᵈ | 6.75 ± 0.29ᵇ | 6.04 ± 0.60ᵇᶜᵈ |
| **4-O-p-Coumaroylquinic acid** | 0.64 ± 0.21ᵃ | 1.13 ± 0.20ᵃᵇ | 1.04 ± 0.14ᵃᵇ | 1.20 ± 0.28ᵃᵇ | 1.29 ± 0.12ᵃᵇ | 1.63 ± 0.21ᵇᶜ | 1.89 ± 0.13ᵇ | 1.63 ± 0.23ᵇᶜ | 1.05 ± 0.06ᵃᵇ | 0.88 ± 0.12ᵃᶜ | 0.90 ± 0.04ᵃᶜ | 1.04 ± 0.09ᵃᵇ | 1.21 ± 0.04ᵃᵇ | 1.28 ± 0.06ᵃᵇ | 1.45 ± 0.07ᵃᵇ | 1.27 ± 0.06ᵃᵇ | 1.45 ± 0.18ᵃᵇ | 1.66 ± 0.41ᵇᶜ |
| **5-O-Caffeoylquinic acid.1** | 61.19 ± 19.78ᵃ | 64.58 ± 19.15ᵃᵇ | 81.57 ± 12.40ᵃᵇ | 80.23 ± 15.68ᵃᵇ | 64.28 ± 15.01ᵃᵇ | 90.13 ± 13.27ᵃᵇ | 124.49 ± 13.58ᵇ | 96.44 ± 12.93ᵃᵇ | 102.06 ± 12.98ᵃᵇ | 78.13 ± 11.73ᵃᵇ | 79.21 ± 2.06ᵃᵇ | 70.31 ± 3.83ᵃᵇ | 95.22 ± 7.26ᵃᵇ | 99.77 ± 5.89ᵃᵇ | 87.16 ± 3.03ᵃᵇ | 88.21 ± 3.97ᵃᵇ | 95.35 ± 15.15ᵃᵇ | 94.09 ± 8.94ᵃᵇ |
| **5-O-Caffeoylquinic acid.2** | 3.92 ± 1.06ᵃ | 5.58 ± 0.88ᵃᵇ | 8.30 ± 0.73ᵇᶜ | 7.41 ± 0.83ᵃᶜ | 6.41 ± 0.63ᵃᶜ | 7.44 ± 1.18ᵃᶜ | 8.14 ± 0.40ᵇᶜᵈ | 8.20 ± 1.38ᵇᶜ | 5.30 ± 0.42ᵃᵇ | 4.45 ± 0.58ᵃᵈ | 4.35 ± 0.16ᵃ | 7.10 ± 0.21ᵃᶜ | 8.59 ± 1.02ᵇᶜ | 8.23 ± 0.33ᵇᶜ | 9.62 ± 0.72ᶜ | 7.11 ± 0.32ᵃᶜ | 6.15 ± 0.20ᵃᶜ | 4.27 ± 0.26ᵃ |
| **5-O-Feruloylquinic acid** | 0.20 ± 0.05ᵃ | 0.51 ± 0.12ᵃᵇ | 0.60 ± 0.15ᵇ | 0.45 ± 0.07ᵃᵇ | 0.40 ± 0.07ᵃᵇ | 0.45 ± 0.04ᵃᵇ | 0.32 ± 0.02ᵃᵇ | 0.30 ± 0.04ᵃᵇ | 0.23 ± 0.01ᵃ | 0.25 ± 0.03ᵃ | 0.20 ± 0.01ᵃ | 0.20 ± 0.01ᵃ | 0.23 ± 0.03ᵃ | 0.43 ± 0.10ᵃᵇ | 0.48 ± 0.07ᵃᵇ | 0.34 ± 0.02ᵃᵇ | 0.50 ± 0.04ᵃᵇ | 0.49 ± 0.04ᵃᵇ |
| **5-O-p-Coumaroylquinic acid** | 1.06 ± 0.33ᵃ | 1.81 ± 0.45ᵃᵇ | 1.80 ± 0.18ᵃᵇ | 1.44 ± 0.19ᵃᵇ | 1.05 ± 0.17ᵃ | 1.41 ± 0.21ᵃᵇ | 1.62 ± 0.24ᵃᵇ | 1.67 ± 0.19ᵃᵇ | 1.76 ± 0.17ᵃᵇ | 1.33 ± 0.21ᵃᵇ | 1.55 ± 0.07ᵃᵇ | 1.52 ± 0.08ᵃᵇ | 1.72 ± 0.09ᵃᵇ | 1.78 ± 0.07ᵃᵇ | 2.00 ± 0.05ᵃᵇ | 1.86 ± 0.04ᵃᵇ | 2.10 ± 0.19ᵇ | 2.08 ± 0.14ᵇ |

**Table S 9:** Concentration of individual hydroxybenzoic acids in haskap pomace samples (mg/kg). Values represent means of four independent repetitions (n = 4). C = control sample; F = frozen sample; P1-P16 = samples treated using pulsed electric field (PEF) under different processing conditions. Mean values followed by different superscript letters within each compound are significantly different according to Tukey’s HSD test (*p* ≤ 0.05).

| **Compound** | **C** | **F** | **P1** | **P2** | **P3** | **P4** | **P5** | **P6** | **P7** | **P8** | **P9** | **P10** | **P11** | **P12** | **P13** | **P14** | **P15** | **P16** |
| --- | --- | --- | --- | --- | --- | --- | --- | --- | --- | --- | --- | --- | --- | --- | --- | --- | --- | --- |
| **Ellagic acid-O-hexoside** | 1.11 ± 0.21ᵃ | 2.04 ± 0.42ᵃᵇ | 2.28 ± 0.23ᵃᵇ | 1.92 ± 0.45ᵃᵇ | 2.12 ± 0.29ᵃᵇ | 2.90 ± 0.35ᵇ | 2.72 ± 0.21ᵇᵈ | 1.53 ± 0.17ᵃᶜᵈ | 1.25 ± 0.08ᵃᶜ | 1.08 ± 0.18ᵃ | 1.32 ± 0.06ᵃᶜ | 1.76 ± 0.20ᵃᵇ | 2.47 ± 0.17ᵇᶜ | 2.41 ± 0.13ᵇᶜ | 2.21 ± 0.21ᵃᵇ | 1.73 ± 0.16ᵃᵇ | 2.19 ± 0.15ᵃᵇ | 2.11 ± 0.16ᵃᵇ |


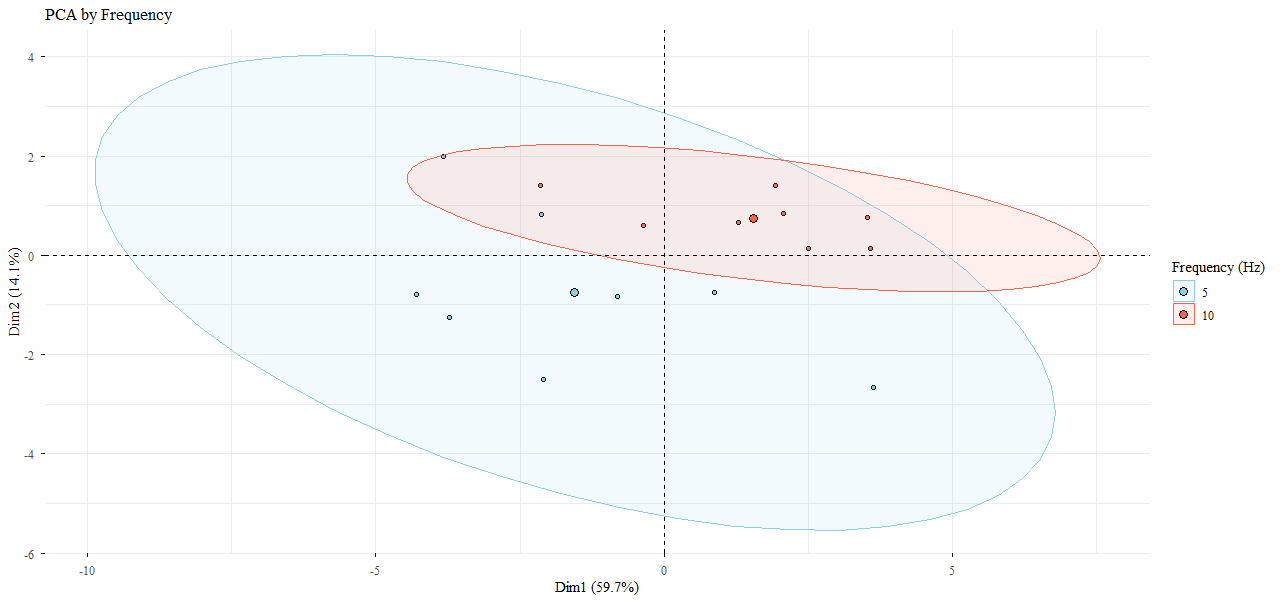


**Figure S 1:** PCA ellipse plot based on pulse frequency


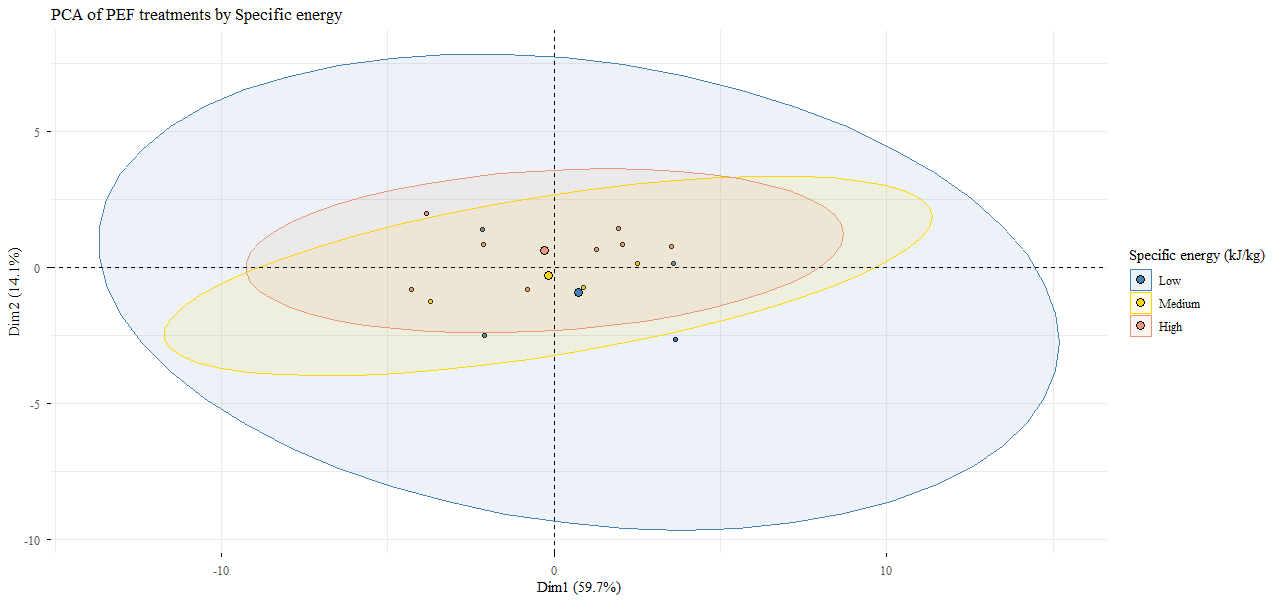


**Figure S 2:** PCA ellipse plot based on specific energy


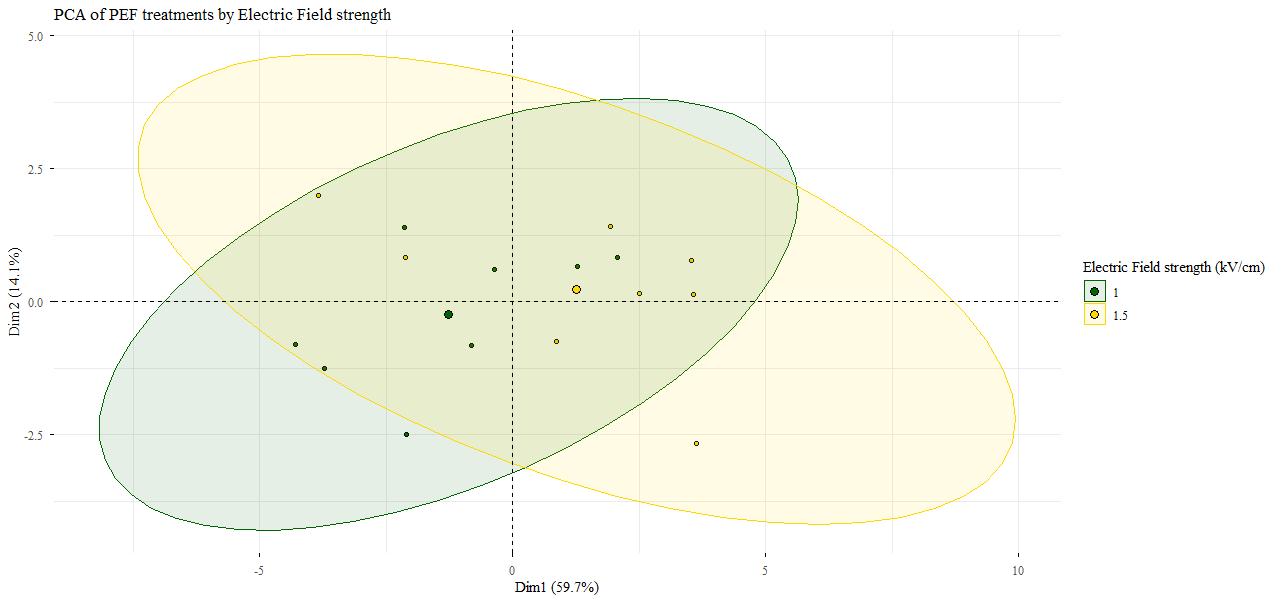


**Figure S 3:** PCA ellipse plot based on electric field strength
